# Circulating Extracellular Vesicle Biomarkers in Chronic Lymphocytic Leukemia: A Preliminary Study on Their Diagnostic, Prognostic, and Predictive Relevance

**DOI:** 10.3390/biom16091293

**Published:** 2026-09-07

**Authors:** Ilaria Laurenzana, Antonella Caivano, Alessio Di Ciancia, Oreste Villani, Maddalena Maietti, Angelo De Stradis, Giovanni D’Arena, Filomena Nozza, Luciana De Luca, Daniela Lamorte

**Affiliations:** 1 Laboratory of Preclinical and Translational Research, IRCCS Centro di Riferimento Oncologico della Basilicata (CROB), via Padre Pio n. 1, 85028 Rionero in Vulture, Italy; ilaria.laurenzana@crob.it (I.L.); alessio.diciancia@crob.it (A.D.C.); f.nozza@oncologico.bari.it (F.N.); daniela.lamorte@crob.it (D.L.); 2 Unit of Clinical Pathology, IRCCS Centro di Riferimento Oncologico della Basilicata (CROB), via Padre Pio n. 1, 85028 Rionero in Vulture, Italy; maddalena.maietti@crob.it; 3 Hematology and Stem Cell Transplantation Unit, IRCCS Centro di Riferimento Oncologico della Basilicata (CROB), via Padre Pio n. 1, 85028 Rionero in Vulture, Italy; oreste.villani@crob.it; 4 Institute for Sustainable Plant Protection, National Research Council (CNR), via G. Amendola n. 122, 70126 Bari, Italy; angelo.destradis@cnr.it; 5 Hematology, “S. Luca” Hospital, ASL Salerno, via Nizza n. 146, 84124 Salerno, Italy; g.darena@aslsalerno.it

**Keywords:** extracellular vesicles, chronic lymphocytic leukemia, biomarkers, B cell antigens, CD200, microRNA, diagnosis, prognosis, prediction

## Abstract

Background/Objectives: Chronic lymphocytic leukemia (CLL) is characterized by marked clinical heterogeneity, underscoring the need for reliable biomarkers to improve diagnosis and risk stratification. Extracellular vesicles (EVs) represent promising liquid biopsy candidates because they reflect the molecular profile of their cells of origin. In CLL, CD200 is a well-established marker of tumor cells; its expression on EVs and its role as a CLL biomarker were poorly characterized. This study investigated the diagnostic and prognostic value of serum EVs in CLL. Methods: Serum EVs were isolated from 83 patients with CLL and 20 healthy subjects (HS). EV morphology, size, and concentration were assessed by transmission electron microscopy and nanoparticle tracking analysis. Flow cytometry was used to evaluate the expression of CD19, CD20, and CD200 on EVs, while digital PCR quantified EV-associated miR-93-5p, miR-125b-5p, miR-150-5p, and miR-484. Results: Compared with HS, CLL patients showed increased total EV counts and higher levels of CD19^+^, CD20^+^, CD200^+^, and CD19^+^/CD20^+^ EVs, together with enhanced CD20 and CD200 expression and reduced miR-93-5p, miR-125b-5p, and miR-484 levels. In contrast, EV-associated miR-150-5p levels were comparable between CLL patients and HS. EV features were correlated with clinical and biological characteristics, including disease stage, cytogenetic abnormalities, treatment, and time to treatment (TTT). Older age was associated with lower EV concentrations but higher CD20 and CD200 expression. Advanced-stage disease was characterized by smaller EVs with increased CD200 expression. Notably, elevated CD200^+^ EV levels and reduced CD19 expression were associated with shorter TTT and earlier treatment initiation. Conclusions: Multiparametric profiling identified distinct quantitative, phenotypic, and molecular features of CLL-derived EVs and revealed CD200 as a novel EV-associated biomarker. These findings support the clinical utility of serum EVs as a new valuable approach for CLL diagnosis, prognosis, and treatment prediction.

## 1. Introduction

Chronic lymphocytic leukemia (CLL) is the most common adult leukemia in Western countries and is characterized by the progressive accumulation of mature B lymphocytes in the peripheral blood, bone marrow, and lymphoid tissues [1]. The diagnosis of CLL is currently based on a combination of hematologic, morphologic, and immunophenotypic criteria. The hallmark is a persistent lymphocytosis, defined as an absolute B-lymphocyte count ≥5 × 10^9^/L for at least three months, often accompanied by characteristic “smudge cells” on blood smear [1]. Flow cytometry is the gold standard for diagnosis of CLL. The typical CLL immunophenotype includes CD5^+^, CD19^+^, CD20^+^, and CD23^+^, with light chain restriction (kappa or lambda). Additionally, CD200 has emerged as an important, robust diagnostic marker, helping differentiate CLL from other B cell lymphoproliferative disorders, like mantle cell lymphoma [2,3]. CD200 is a glycoprotein expressed on various cell types, including B and T lymphocytes, dendritic cells, and endothelial cells [4]. Different studies have demonstrated that CD200 is overexpressed in CLL cells, and its soluble ectodomain (sCD200) is detectable in the serum of CLL patients. Notably, baseline serum levels of sCD200 have been identified as a prognostic factor in CLL, with higher levels correlating with adverse clinical outcomes [5].

In addition to immunophenotypic analysis, molecular alterations play a key role in the risk stratification and prognosis of CLL. The most clinically relevant alterations include IGHV (immunoglobulin heavy chain variable region) TP53 deletion, Notch receptor 1 (NOTCH1) mutations, and other cytogenetic abnormalities, such as del(13)(q14), trisomy 12, and del(11)(q22–q23) (ATM gene deletion) [6]. These markers are routinely integrated with clinical staging systems (Rai or Binet), which stratify patients based on lymphocytosis, lymphadenopathy, organomegaly, anemia, and thrombocytopenia [1]. In recent years, with the advent of targeted therapies, the therapeutic landscape of CLL has changed, improving patient outcomes and modifying the natural history of the disease. In this context, traditional Rai and Binet staging systems show important limitations in predicting disease courses and treatment responses. Consequently, there is an increasing need to integrate them with new prognostic biomarkers because of the high variability in disease course, as some patients with CLL classified as having early-stage disease could rapidly progress if they carry other high-risk features [1].

In this context, extracellular vesicles (EVs) have recently emerged as promising biomarkers in CLL. EVs are lipid bilayer-enclosed particles released by all cell types, carrying proteins, lipids, and nucleic acids that reflect the phenotype and functional state of their cell of origin [7,8]. In CLL, leukemic B cells have been shown to release large amounts of EVs, which may participate in reshaping the tumor microenvironment, promoting immune evasion, and facilitating disease progression. CLL-derived EVs express B cell-associated markers like CD19 and CD20, suggesting that they could serve as a “fingerprint” of the origin clone [9,10,11]. In addition, tumor-derived EVs have been reported to express immune-regulatory molecules, including CD200, which may contribute to immune suppression through interaction with CD200R on immune cells [12].

Regarding EV cargo, microRNAs (miRNAs) play a key role in regulating gene expression and can influence apoptosis, proliferation, and B cell receptor signaling in CLL. Thanks to their encapsulation in EVs, they are particularly stable in circulation and can thus be reliably quantified in peripheral blood samples. Specific miRNAs, including miR-125b, miR-150, miR-93-5p, and miR-484, have been reported to be dysregulated in tumors, and their levels in EVs may provide useful diagnostic and prognostic information with a minimally invasive methodological approach [13,14,15,16].

Taken together, these findings highlight the dual role of EVs in CLL as both active mediators of tumor biology and a potential source of clinically promising biomarkers.

In this study, we performed a comprehensive characterization of serum-derived EVs from CLL patients and healthy subjects, evaluating EV concentration, size, surface marker profile, and miRNA cargo. Furthermore, we investigated the association between EV features and clinical parameters to explore their potential diagnostic, prognostic, and predictive significance.

The novelty of our study was to identify the EV characteristics, in particular the new EV-associated CD200 and miRNAs, and to provide preliminary, additional, and complementary information which could be relevant, in the future, to disease biology, risk stratification, and clinical management.

## 2. Materials and Methods

### 2.1. Serum Samples and CLL Cell Line

Peripheral blood (PB) samples were obtained at diagnosis from 83 CLL patients recruited between 2012 and 2022 and from 20 healthy subjects (HS) matched for age (41–80 years old), gender, and lifestyle. Patients were followed up for 200 months. Patients’ clinicopathological characteristics are described in Table 1. This study complied with the international Helsinki Declaration regulation for research on human subjects and was approved by the Basilicata Ethical Committee (Prot. N. TS/CEUR 20200050581). Written informed consent was obtained from all subjects.

To obtain serum, three mL of PB was drawn into Vacutainer SST II Advance tubes (Becton Dickinson, BD, Franklin, NJ, USA). PB samples were processed within 2 h of collection. Serum was obtained by centrifugation at 3220× *g* for 10 min at +4 °C. Hemolysis was assessed by visual inspection, and samples exhibiting visible red coloring were excluded from further analysis. Serum aliquots were stored at −80 °C until use. To preserve EV integrity, samples underwent a single freeze–thaw cycle and were thawed only once immediately before EV isolation.

The CLL HG-3 cell line (ACC 765, DSMZ, Braunschweig, Germany) was cultured in RPMI 1640 medium (Gibco, Life Technologies, Carlsbad, CA, USA) supplemented with 10% FBS (Gibco) and 1% penicillin-streptomycin (Gibco) at 37 °C and 5% CO_2_.

### 2.2. EV Isolation

EV isolation was performed as previously reported [17]. Briefly, 500 μL of serum (or 200 μL for EV-RNA extraction) was centrifuged using a bench centrifuge (MicroCL 21R centrifuge, ThermoFisher Scientific, Wilmington, DE, USA) at 200× *g* for 5 min at 4 °C. Supernatant was then centrifuged at 14,300× *g* for 1 h at 4 °C. The resulting pellet was washed with 0.22 μm filtered phosphate-buffered saline (PBS) without calcium and magnesium salts (Gibco), centrifuged at 14,300× *g* for 1 h at 4 °C, and resuspended in 500 μL of 0.02 μm filtered PBS.

According to MISEV 2023 guidelines [7], the isolation of EVs was confirmed by analyzing morphology, quantity, and size using subsequent methods as also reported in our Patent No. 102023000020943 [18].

To obtain cell line-derived EVs, 23 × 10^6^ HG-3 cells were cultured at a density of 1.2 × 10^6^ cells/mL of RPMI 1640 medium without FBS for 48 h. Supernatant was collected for the next analysis; EVs were obtained as reported above and resuspended in 1 mL of 0.02 μm filtered PBS.

### 2.3. Transmission Electron Microscopic Analysis

Seum EV morphology was visualized by a transmission electron microscope (TEM) as previously reported [19]. Specifically, 20 μL of EV sample suspension was applied to a Pioloform-coated nickel grid (200 mesh; TAAB Laboratories Equipment Ltd., Aldermaston, UK). The grid was floated for 2 min on the sample drop and rinsed in a 20 μL double-distilled water drop. Negative staining was performed with 200 μL of 1% *w*/*v* UA-zero EM stain solution (Agar-Scientific Ltd., Stansted, UK). After draining the excess staining solution, the specimen was examined in a Philips Morgagni 282D TEM, operating at 60 kV. Electron micrographs of negatively stained samples were photographed on a Kodak electron microscope film 4489 (Kodak Company, Rochester, NY, USA).

### 2.4. Nanoparticle Tracking Analysis

EV concentration and size distribution were defined by Nanoparticle Tracking Analysis (NTA) using NanoSight NS300 (Malvern Panalytical Ltd., Malvern, UK), as previously reported [17]. Briefly, immediately after isolation, EV samples were diluted in a final volume of 400 μL. Five videos of 60 s each per sample were obtained in light scattering mode as indicated in the manufacturer’s protocol. All data were processed using NTA 3.2 software. EV concentration, mean, mode, and D10 and D90 values were reported.

### 2.5. Flow Cytometric Analysis of CLL Cell Line and EV Samples

Flow cytometric analysis was performed on DxFlex and analyzed by Kaluza C 1.2.1 software (Beckman Coulter, Brea, CA, USA). To prepare the flow cytometer for EV analysis, Megamix-Plus FSC (Biocytex, Marseille, France) was used to set up the instrument for physical parameters: forward scatter, FSC, and side scatter, SSC (18). EV samples were labeled with 5-carboxyfluorescein diacetate-succinimidyl ester (CFDA-SE, Sigma-Aldrich, Darmstadt, Germany) as previously reported [17]. Notably, this dye becomes fluorescent upon esterase-mediated conversion to carboxyfluorescein succinimidyl ester (CFSE). CFSE fluorescence was used as a readout of EV integrity, as esterase activity is preserved in intact vesicles. To set the CFSE-positive EV gate, 0.02 μm filtered PBS and unstained EVs were used [19].

To characterize the EV phenotype and establish the correct gating strategy, HG-3 cells and derived EVs were used as a technical control. Although HG-3 is EBV-positive, this CLL cell line is positive for CD19 and CD20; thus, it was used exclusively for assay setup. Before EV isolation, the HG-3 cell line was verified for surface marker expression. In brief, after being cultured without FBS for 48 h, 80,000 harvested cells were washed with PBS and incubated with allophycocyanin (APC) anti-CD19 (clone SJ25C1, BD), phycoerythrin (PE) anti-CD20 (clone L27, BD), and PE anti-CD200 (clone 552475, BD) monoclonal antibodies at room temperature for 15 min in the dark. After incubation, 10,000 events were acquired. In accordance with the cell datasheet, HG-3 cells resulted positive for CD19, CD20, and CD200 (Figure S1A).

EVs isolated from HG-3 were labeled with CFDA-SE and incubated for 40 min at 37 °C with 100 ng of APC anti-CD19 and PE anti-CD20, or PE anti-CD200, and their respective isotype controls. A total of 50,000 events were acquired at a low flow rate. HG-3-derived EVs expressed all three markers, confirming their cellular origin and the retention of immunophenotypic features characteristic of CLL cells (Figure S1B).

To quantify CLL patient-derived EVs, after CFDA-SE staining and monoclonal antibody labeling, 3 μL of EV samples were transferred to TruCount tubes (BD) containing 400 μL of 0.02 μm filtered PBS. A total of 50,000 events were acquired at a low flow rate. The quantity of EVs (EVs/mL) positive for specific antigen was calculated with the formula previously reported [17].

### 2.6. EV RNA Isolation, Quantification, Reverse Transcription, and EV miRNA Analysis

EV miRNA analysis was performed on 20 HS and 20 CLL patients. CLL patients were selected to be representative of the overall study cohort with respect to the main clinical and biological characteristics, including age, disease stage, and treatment status (Table S1).

Total EV RNA was isolated according to our Patent No. 102023000020943 [18], and then it was immediately quantified by a Qubit 4.0 Fluorometer (ThermoFisher Sc.) using the Qubit microRNA assay kit (Life Technologies). cDNA was synthesized using the TaqMan Advanced miRNA cDNA synthesis kit (Applied Biosystems, Foster City, CA, USA), following manufacturer’s instructions, starting from a precise EV-RNA amount.

Four miRNA (miR) levels, including miR-93-5p, miR-125b-5p, miR-150-5p, and miR-484, were quantified by a QX200 droplet digital PCR (ddPCR) system (Bio-Rad Laboratories, Hercules, CA, USA), as previously reported [19]. For each miRNA, 10 μL of 1:1000 diluted cDNA was used. Cycling conditions on a Veriti thermal cycler (Applied Biosystem) were 95 °C for 10 min, then 45 cycles of 95 °C for 15 s and 58 °C for 1 min, and finally 98 °C for 10 min and 4 °C infinite hold. A ramping rate of 2 °C/s was used in every step. All plates were read in the Droplet Reader and analyzed by QuantaSoft TM version 1.7.4 software (Bio-Rad). For each sample, the number of copies/μL generated by software analysis was multiplied by the used cDNA dilution factor to obtain the number of copies/μL.

### 2.7. Systematic Analysis of EV microRNAs

The analysis of putative target genes was performed using miRTargetLink 2.0. Specifically, this tool was used in unidirectional mode, selecting “Homo sapiens” as species and “Target-gene overlap between multiple microRNAs” as miRNA-centric search. All four miRNAs have been inserted. To obtain a more precise network, we selected “strong validated miRNA targets”. The resulting miRNA target genes were used for enrichment analysis by ShinyGO 0.77 [ShinyGO 0.77 (sdstate.edu)], selecting different pathway databases, including GO Biological Process, KEGG, Curated.Panther, and Curated.WikiPathways, with a 0.05 FDR cut-off and a 20–5000 pathway size.

### 2.8. Statistical Analysis

All statistical analyses were performed using GraphPad Prism 10.6.1 software. Comparisons between two independent groups were performed using the non-parametric Mann–Whitney U test. Data are presented as the median with 95% confidence intervals.

For comparisons showing statistically significant differences between two groups, Receiver Operating Characteristic (ROC) curve analyses were performed to evaluate the diagnostic ability of EV parameters and to identify the optimal cut-off values for discriminating between groups.

Comparisons among three groups (Rai or Binet stage groups) were performed using the Kruskal–Wallis test.

Correlations between patient age or absolute lymphocyte counts and EV parameters were assessed using Spearman’s rank correlation analysis. Spearman’s correlation coefficient (r_s_), the corresponding 95% confidence interval, and the two-tailed *p*-value were calculated.

Time to treatment (TTT) was analyzed using Kaplan–Meier curves. Patients were stratified into two groups according to the ROC-derived cut-off values for CD200^+^ EVs/µL and CD19 Mean Fluorescence Intensity (MFI). Differences between Kaplan–Meier curves were assessed using the log-rank (Mantel–Cox) test.

All statistical tests were bilateral, and a *p*-value of <0.05 was considered statistically significant. Given the exploratory nature of the study and the limited cohort size, no correction for multiple comparisons was applied. Therefore, the reported *p*-values should be interpreted as exploratory and hypothesis-generating, and the observed associations will require validation in larger independent cohorts.

## 3. Results

### 3.1. Patients’ and Healthy Subjects’ Clinical Characteristics

Serum samples were collected from 83 patients with CLL (median age 66 years; range 41–89; 51 males and 32 females) and 20 healthy subjects (HS, median age 68 years; range 39–88; 10 males and 10 females). The median follow-up of CLL patients was 54 months (range 1–273 months).

The main biological and clinical characteristics of the CLL cohort, including Rai stage, Binet stage, karyotype, and treatment status, are summarized in Table 1. Additional prognostic biomarkers, including IGHV mutational status, TP53 mutation/deletion, NOTCH1 mutations, and β2-microglobulin levels, were not available for all patients. Therefore, these variables were not included in the statistical analysis because of the limited sample size.

### 3.2. Characterization of EVs Isolated from Healthy Subjects and CLL Patients

EVs were isolated from the serum of 83 CLL patients and 20 HS. EV morphology, size distribution, and particle integrity were assessed using three complementary techniques.

**Figure 1:**
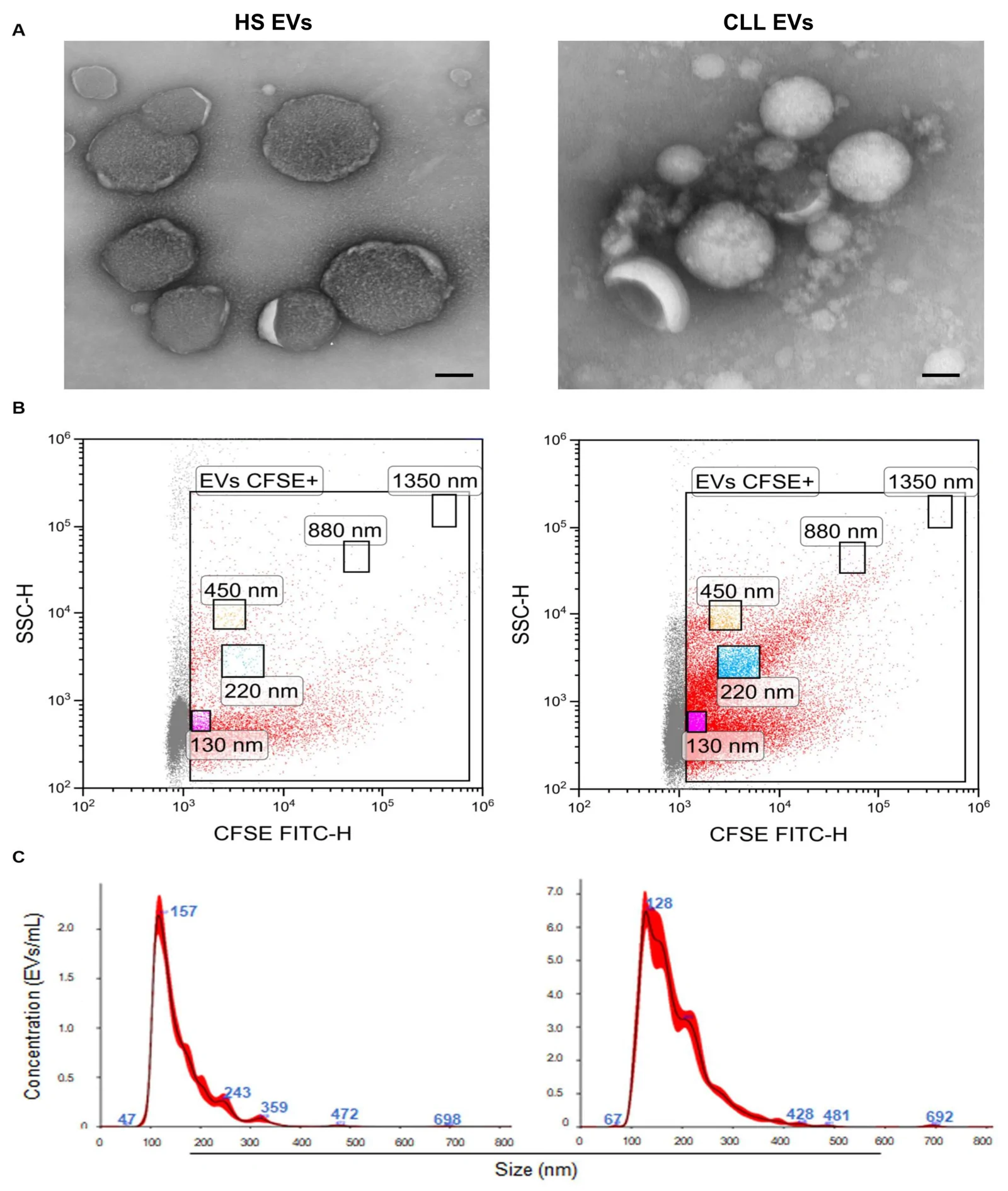
Characterization of EVs from a representative healthy subject (HS) and CLL patients. (**A**) TEM photos of HS- and CLL-derived EVs (bars indicate 100 nm). (**B**) Flow cytometer CFSE-H/SSC-H dot plots of HS- and CLL-derived EVs. Little squares and their relative number (nm) represent the beads’ sizes; the largest square represents gated events corresponding to CFSE-positive (CFSE^+^) EVs, which represent intact EVs in a size range of 130–1350 nm. (**C**) NTA size distribution profiles of EVs from HS and CLL patients.

Firstly, TEM analysis was performed on representative samples to evaluate EV morphology (Figure 1A). In particular, HS samples exhibited EVs with a bilayer membrane containing a homogeneous matrix both in composition and in distribution within them. Their average diameter was approximately 200 nm, ranging from 100 to 300 nm. Instead, CLL-derived EVs structurally have a more globoid shape and contain a heterogeneous matrix, the larger ones with a zonal condensation of their compounds, unlike the smaller ones with a homogeneous matrix. The average CLL-EV diameter was approximately 100 nm, with a range of 50–150 nm (Figure 1A).

To further assess membrane integrity, EV pellets were labeled with the membrane-permeant non-fluorescent CFDA-SE and analyzed by flow cytometry. As shown in Figure 1B, both HS and CLL EV pellets were enriched in CFSE-positive particles, indicating the presence of intact membrane-bound vesicles capable of internalizing CFDA-SE and converting it into fluorescent CFSE. Flow cytometric analysis showed EVs with a diameter range of 130–1350 nm both in HS and in CLL patients (Figure 1B). In addition, NTA analysis further confirmed the presence of EVs with a heterogeneous size profile with a dimensional range of 47–700 nm, with the majority of particles measuring between 45 and 200 nm in both HS and CLL samples (Figure 1C).

Overall, the diameter of EVs determined by TEM, NTA, and flow cytometry was consistent with the expected dimensions of small EVs.

As previously demonstrated, our EV isolation protocol yields highly purified EV pellets devoid of detectable serum contaminants. Quantification of total proteins, albumin, HDL, LDL, VLDL, ApoA1, and ApoB in serum, EV pellets, and the corresponding supernatants confirmed that these contaminants remained in the supernatant and were not co-isolated with EVs, validating the purity of our EV preparations [17,19].

### 3.3. CLL Patients Exhibited Higher EV Concentration and a Smaller EV Size than HS

**Figure 2:**
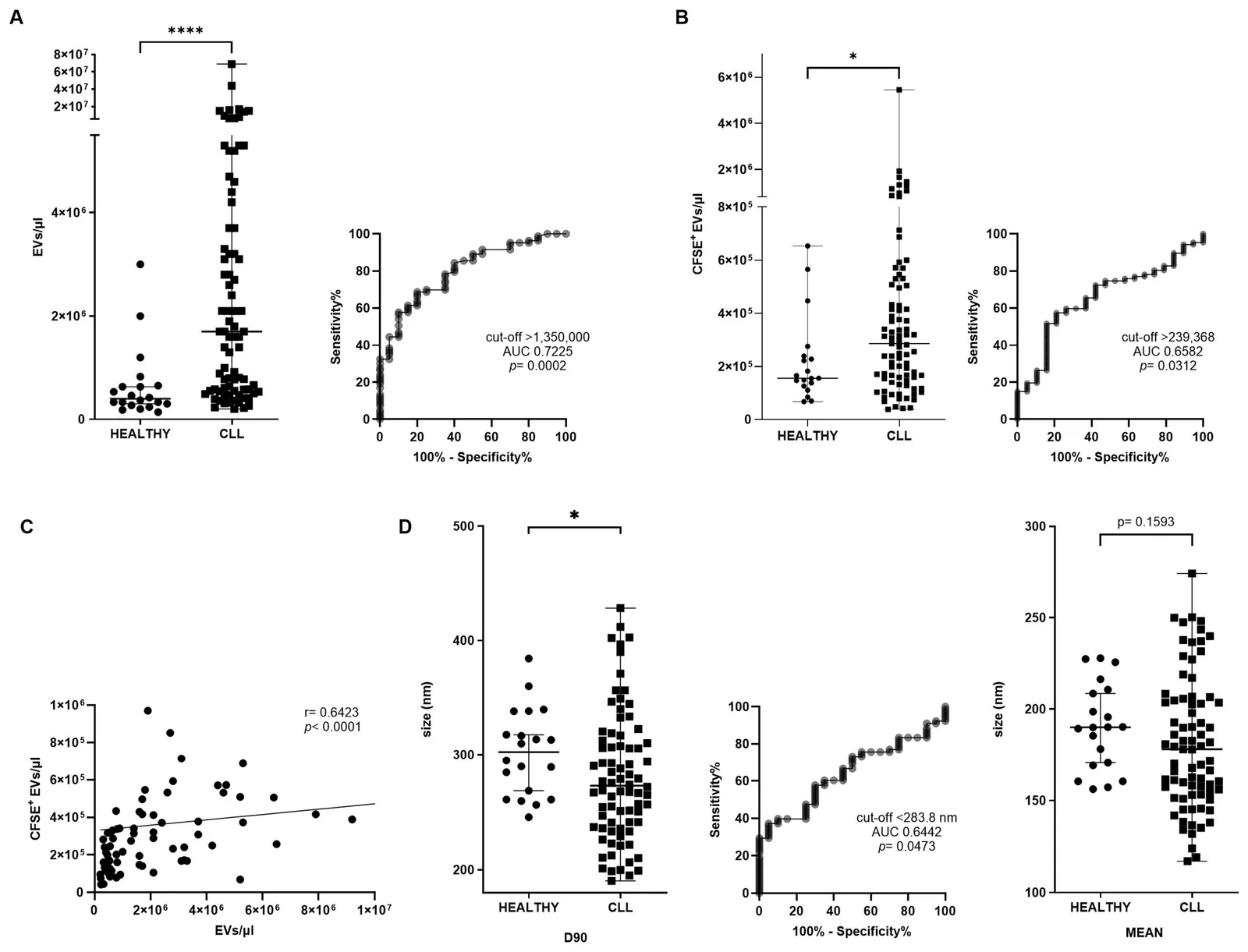
Concentration and size comparison between EVs from HS and CLL patients. Dot plot and ROC curve analysis of (**A**) NTA concentration (EVs/µL) and (**B**) flow cytometric concentration (CFSE^+^ EVs/µL) of comparison between HS and CLL patients. (**C**) Correlation plot of NTA (EVs/µL) and flow cytometric EV concentration (CFSE^+^ EVs/µL) analysis. (**D**) Dot plot of D90 (nm) and mean (nm), and D90 ROC curve comparison between HS and CLL patients. Each symbol represents a single sample, and horizontal bars represent median values with a 95% confidence interval (CI). Statistically significant analyses are indicated by asterisks: **** *p* < 0.0001, * *p* < 0.05.

Comparison between the 83 CLL patients and 20 HS by NTA showed a significantly higher EV concentration in CLL patients than in HS (median, 1.7 × 10^6^ vs. 3.95 × 10^5^ EVs/µL, *p* < 0.0001; Figure 2A). ROC curve analysis identified a cut-off value of 1.35 × 10^6^ EVs/µL for discriminating CLL patients from HS (AUC 0.7225; *p* = 0.0002, sensitivity 56.63%, and specificity 90%). Flow cytometric analysis confirmed these findings, showing significantly higher EV concentration in CLL patients than in HS (median, 2.86 × 10^5^ vs. 1.55 × 10^5^ EVs/µL, *p* = 0.03; Figure 2B). Cut-off value established by ROC analysis was 2.3 × 10^5^ EVs/µL (AUC 0.6582; *p* = 0.0312, sensitivity 57.47%, and specificity 78.95%).

A significant positive correlation was observed between EV counts obtained by flow cytometry and NTA (r = 0.6423, *p* < 0.0001), indicating good agreement between the two quantification methods (Figure 2C).

Analysis of EV size by NTA showed that CLL-derived EVs were smaller than HS-derived ones. Specifically, the D90 value was significantly lower in CLL-derived EVs (median, 273.9 vs. 302.4 nm; *p* = 0.05; Figure 2D). ROC curve analysis identified an optimal D90 cut-off value of 283.8 nm (AUC 0.6442; *p* = 0.0473, sensitivity 56.96%, and specificity 70%). The mean EV diameter was also lower in CLL-derived EVs than in HS-derived EVs (median, 178 vs. 190 nm), although this difference did not reach statistical significance (*p* = 0.1593; Figure 2D).

Similarly, D10 and mode values tended to be lower in CLL samples; however, these differences were not statistically significant (Figure S2).

### 3.4. CLL Patients Display Increased Levels of EVs Positive for Disease-Specific Surface Markers

To identify CLL-specific EVs, we evaluated the expression of the B cell-typical markers CD20 and CD19, their co-expression, and CD200 by flow cytometry. All of these markers, both single positive and double positive for CD19^+^/CD20^+^, were detected on EVs isolated from the HG-3 CLL cell line (Figure S1), confirming their association with CLL-derived vesicles.

**Figure 3:**
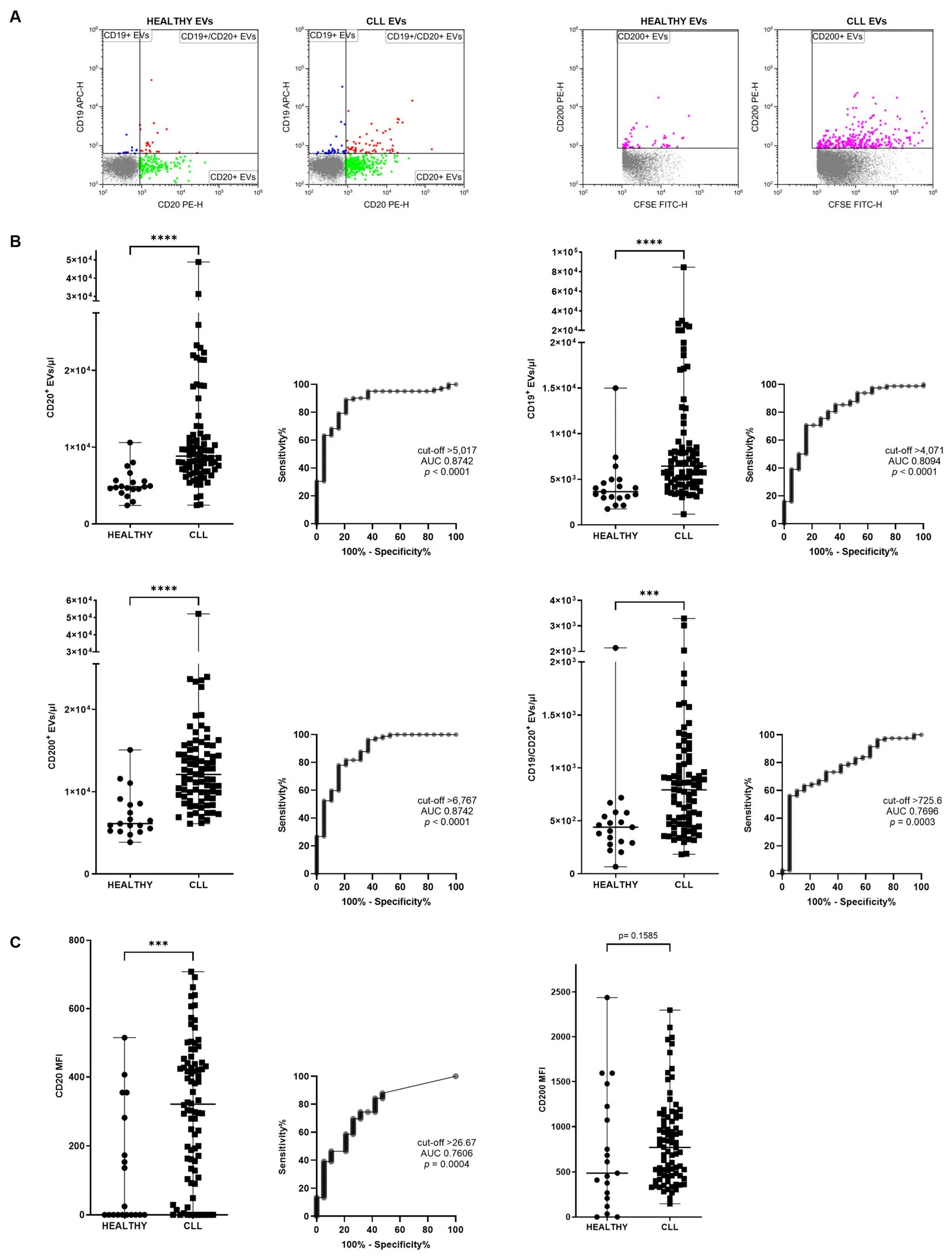
Comparison analysis of flow cytometric data of EVs from HS and CLL. (**A**) Representative dot plots of HS- and CLL-EVs labeled with CD19, CD20 (**on the left**) and CD200 (**on the right**). Dot plot and ROC curve comparisons of (**B**) number of CD20^+^, CD19^+^, CD200^+^, and CD19^+^/CD20^+^ EVs/µL and of (**C**) CD20 and CD200 MFI in HS versus CLL samples. Each symbol represents a single subject, and horizontal bars represent median values with a 95% CI. Statistically significant analyses are indicated as values or by asterisks: **** *p* < 0.0001, *** *p* < 0.001.

As shown in Figure 3A, CD20^+^, CD19^+^, CD200^+^, and CD19^+^/CD20^+^ EVs were detected in all serum samples from both CLL patients and HS. Interestingly, all four EV subpopulations were significantly more abundant in CLL patients than in HS. Specifically, the median concentration of CD20^+^ EVs was 8833 vs. 4841 EVs/µL (*p* < 0.0001), CD19^+^ EVs 6445 vs. 3642 EVs/µL (*p* < 0.0001), CD200^+^ EVs 12,090 vs. 6116 EVs/µL (*p* < 0.0001), and CD19^+^/CD20^+^ EVs 793.5 vs. 439.5 EVs/µL (*p* = 0.0002) in CLL patients and HS, respectively (Figure 3B).

ROC curve analysis identified the following optimal cut-off values for discriminating CLL patients from HS: >5017 CD20^+^ EVs/µL (AUC = 0.8742, sensitivity 95.40%, specificity 63.16%, *p* < 0.0001), >4071 CD19^+^ EVs/µL (AUC = 0.8094, sensitivity 85.06%, specificity 63.16%, *p* < 0.0001), >6767 CD200^+^ EVs/µL (AUC = 0.8742, sensitivity 95.40%, specificity 63.13%, *p* < 0.0001), and >725.6 CD19^+^/CD20^+^ EVs/µL (AUC = 0.7696, sensitivity 54.02%, specificity 94.74%, *p* = 0.0003) (Figure 3B).

Additionally, by evaluating the expression level of these markers on EVs by measuring the MFI, we observed a significantly higher MFI of CD20 in CLL EVs than in HS ones (median of 320.8 vs. 0, respectively, *p* = 0.0003; Figure 3C). ROC curve analysis confirmed the discriminatory ability of CD20 MFI, identifying an optimal cut-off value of >26.76 (AUC 0.7606, 84.15% sensitivity and 57.89% specificity, *p* = 0.0004; Figure 3C). A similar trend was observed for CD200 MFI, which was higher in CLL EVs than HS EVs (median, 770.3 vs. 485.7, *p* = 0.1585; Figure 3C). No significant differences in CD19 MFI were detected between the two groups (Figure S3).

### 3.5. CLL Patients Revealed Decreased Expression of EV miR-93-5p, miR-125b-5p, and miR-484 Compared with Healthy Subjects

Circulating miRNAs have emerged as promising biomarkers for cancer diagnosis, prognosis, and therapeutic response. Based on this evidence, we investigated a panel of serum EV-associated miRNAs implicated in cancer, including miR-93-5p, miR-125b-5p, miR-150-5p, and miR-484.

**Figure 4:**
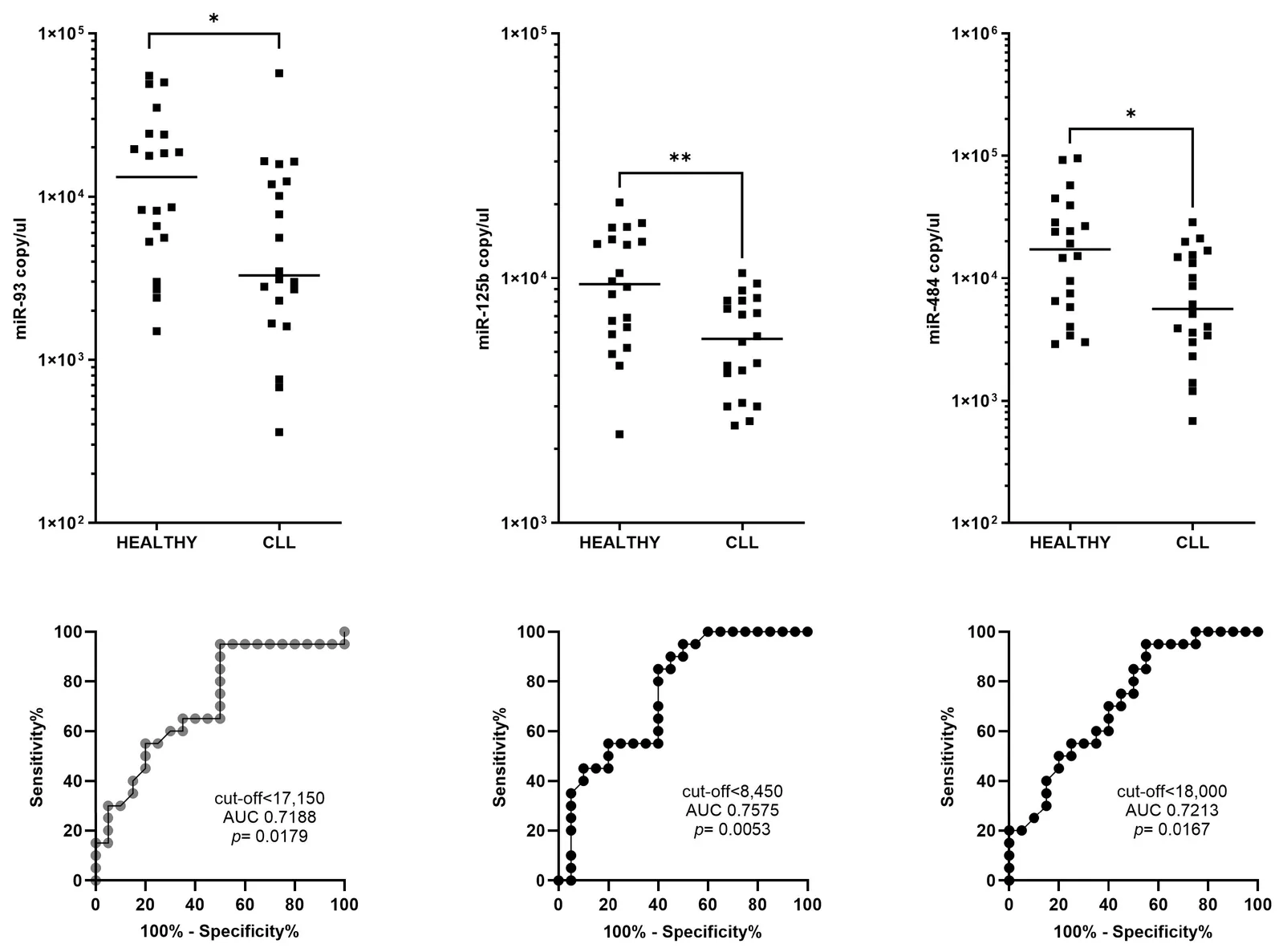
Analysis of EV-miRNA expression in HS vs. CLL. Dot plot ROC curve of EV-miR-93-5p, miR-125b-5p, and miR-484 expression, as copies/µL, comparisons between HS and CLL. Each symbol represents a single subject, and horizontal bars represent median values with a 95% CI. Statistically significant analyses are indicated by asterisks: * *p* < 0.05, ** *p* < 0.01.

All four miRNAs were detectable in EVs isolated from both CLL patients and HS, although their expression levels differed between the two groups (Figure 4 and Figure S4).

As shown in Figure 4, EV-associated miR-93-5p, miR-125b-5p, and miR-484 were significantly down-regulated in CLL patients compared with HS. Specifically, the median expression of miR-93-5p was 8809 vs. 18,230 copies/µL (*p* = 0.05), that of miR-125b-5p was 5895 vs. 10,305 copies/µL (*p* = 0.0013), and that of miR-484 was 9184 vs. 26,240 copies/µL (*p* = 0.01) in CLL patients and HS, respectively. In contrast, no significant difference was observed in EV-associated miR-150-5p expression between the two groups (median, 53,165 vs. 49,775 copies/µL; *p* = 0.8; Figure S4). ROC curve analysis demonstrated that the expression levels of miR-93-5p, miR-125b-5p, and miR-484 were able to discriminate CLL patients from HS. The optimal cut-off values were <17,150 copies/µL for miR-93-5p (AUC 0.7188, 95% sensitivity and 50% specificity, *p* = 0.0179), <8450 copies/µL for miR-125b-5p (AUC 0.7212, 85% sensitivity and 60% specificity, *p* = 0.0053), and <18,000 copies/µL for miR-484 (AUC 0.7575, 85% sensitivity and 50% specificity, *p* = 0.0167) (Figure 4).

To explore the potential functional role of these EV-associated miRNAs in CLL, we investigated their predicted mRNA targets, identifying a total of 178 genes (Table S2).

**Figure 5:**
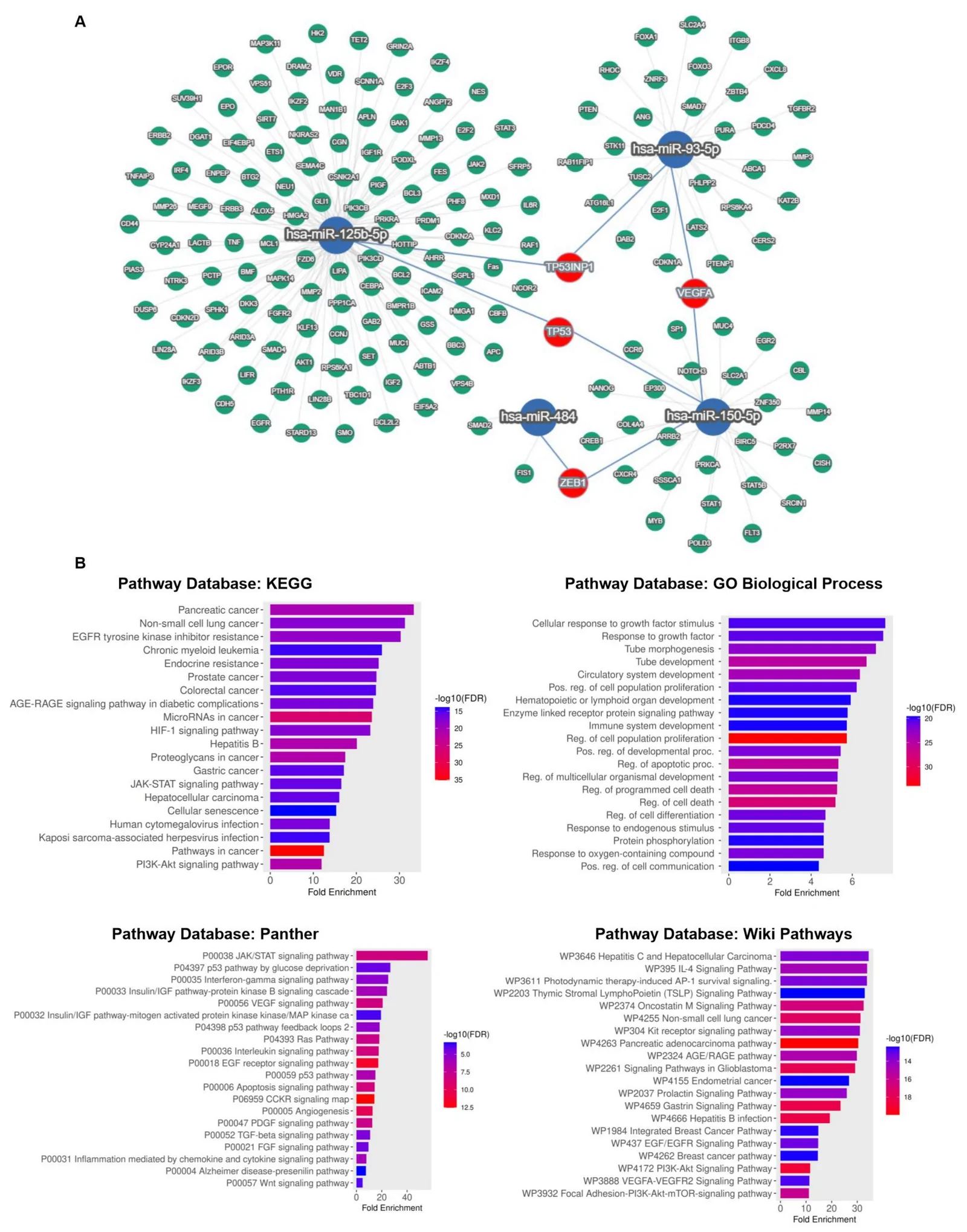
Systematic analysis of EV-miRNA targets. (**A**) Random network layout obtained from miRTargetLink 2.0 indicating only strongly validated target genes of miR-93, miR-125b, miR-150, and miR-484. The target genes of two miRNAs are indicated in the red circle. (**B**) Pathway analysis of miRNA target genes using KEGG, GO Biological Process, Panther, and WikiPathways databases.

As shown in Figure 5A, several biologically relevant targets were identified, including *TP53*, *TP53INP1*, *ZEB1*, and *VEGF*.

Gene ontology enrichment analysis of the predicted targets identified “Pathways in Cancer” as the most significantly enriched KEGG pathway. Additional enrichment analysis also highlighted the “MicroRNAs in Cancer” pathway (Figure 5B). GO Biological Process analysis indicated that the predicted target genes are primarily involved in the regulation of cell proliferation, apoptosis, and cell death. Furthermore, Panther and WikiPathways enrichment analyses identified significant enrichment of the STAT and p53 signaling pathways (Figure 5B).

### 3.6. Association Between EV Characteristics and Clinical Features of CLL Patients

To preliminarily assess the potential clinical relevance of EVs in CLL, we analyzed the association between EV characteristics and selected clinical parameters, including age, Rai stage, Binet stage, and FISH cytogenetic profile.

**Figure 6:**
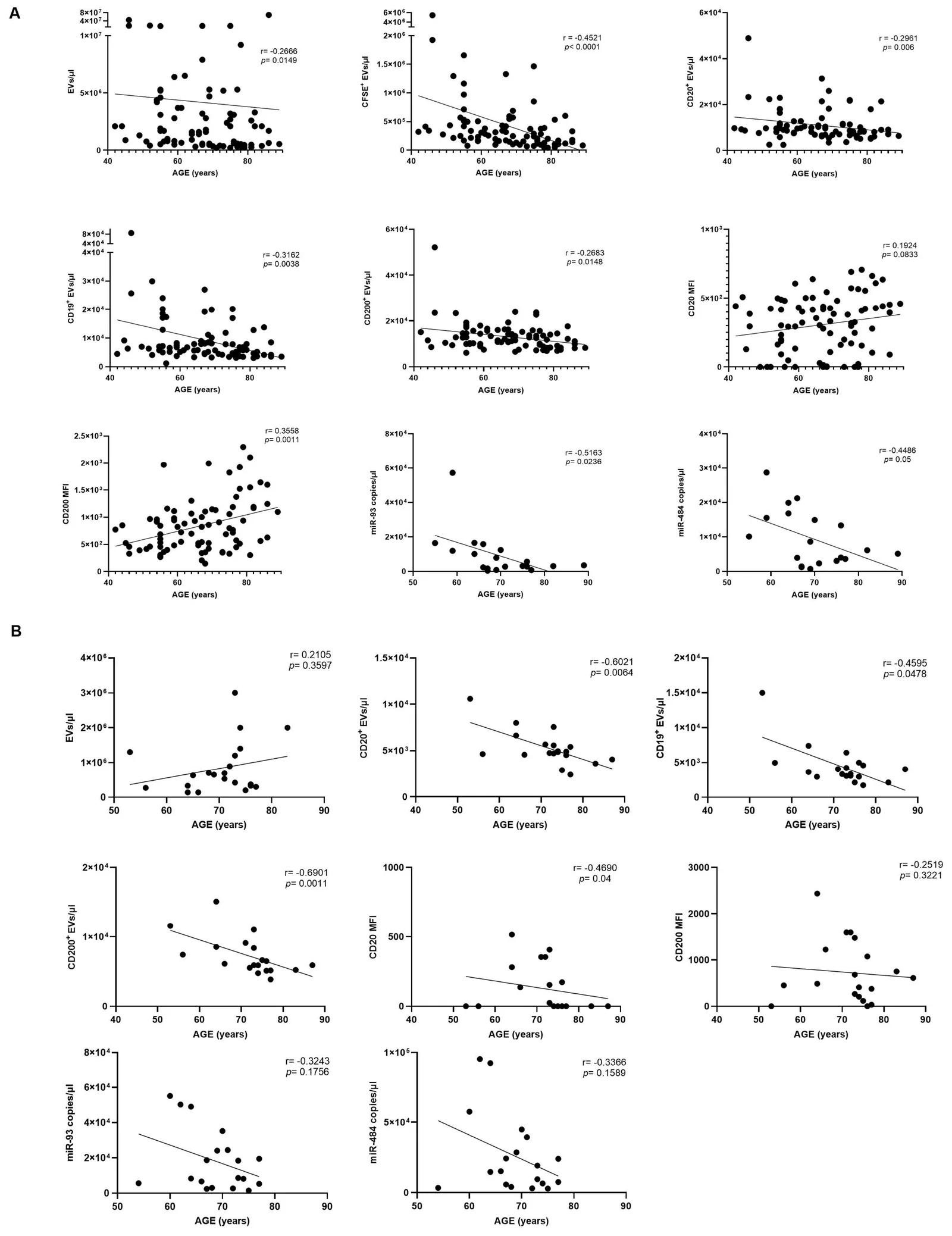
Association of EV parameters with age of CLL patients (**A**) and HS (**B**). Correlation of EV concentration by NTA (EVs/µL) and by flow cytometry (CFSE^+^ EVs/µL) of CD20^+^ EVs/µL, CD19^+^ EVs/µL, CD200^+^ EVs/µL, CD20 MFI, CD200 MFI, miR-93-5p miR-484 (copies/µL), and patient age (years). Each symbol represents a single patient; r and *p* are reported on graphs.

Correlation analysis revealed a significant inverse association between patient age and EV concentration (Figure 6). Specifically, EV counts decreased with increasing age, as measured by both NTA (r = −0.2666, *p* = 0.0149) and flow cytometry (r = −0.4521, *p* < 0.0001). A similar inverse correlation with age was observed for CD20^+^ EVs/µL (r = −0.2961, *p* = 0.006), CD19^+^ EVs/µL (r = −0.3162, *p* = 0.0038), CD200^+^ EVs/µL (r = −0.2683, *p* = 0.0148), EV-associated miR-93-5p (r = −0.5163, *p* = 0.0236), and miR-484 (r = −0.4486, *p* = 0.05). In contrast, the expression levels of CD20 and CD200 on EVs, evaluated as MFI, showed a positive correlation with age. This association reached statistical significance for CD200 MFI (r = 0.3558, *p* = 0.0011), whereas only a trend was observed for CD20 MFI (r = 0.1924, *p* = 0.0833) (Figure 6).

Correlation analysis performed in HS showed a positive association between age and EV concentration, in contrast to the inverse correlation observed in CLL patients (Figure 6).

No significant correlations were found in both CLL and HS samples between age and EV size parameters (D10, D90, mean, and mode), CD19^+^/CD20^+^ EVs/µL, CD19 MFI, EV-associated miR-125b-5p, and miR-150-5p (Figure S5).

**Figure 7:**
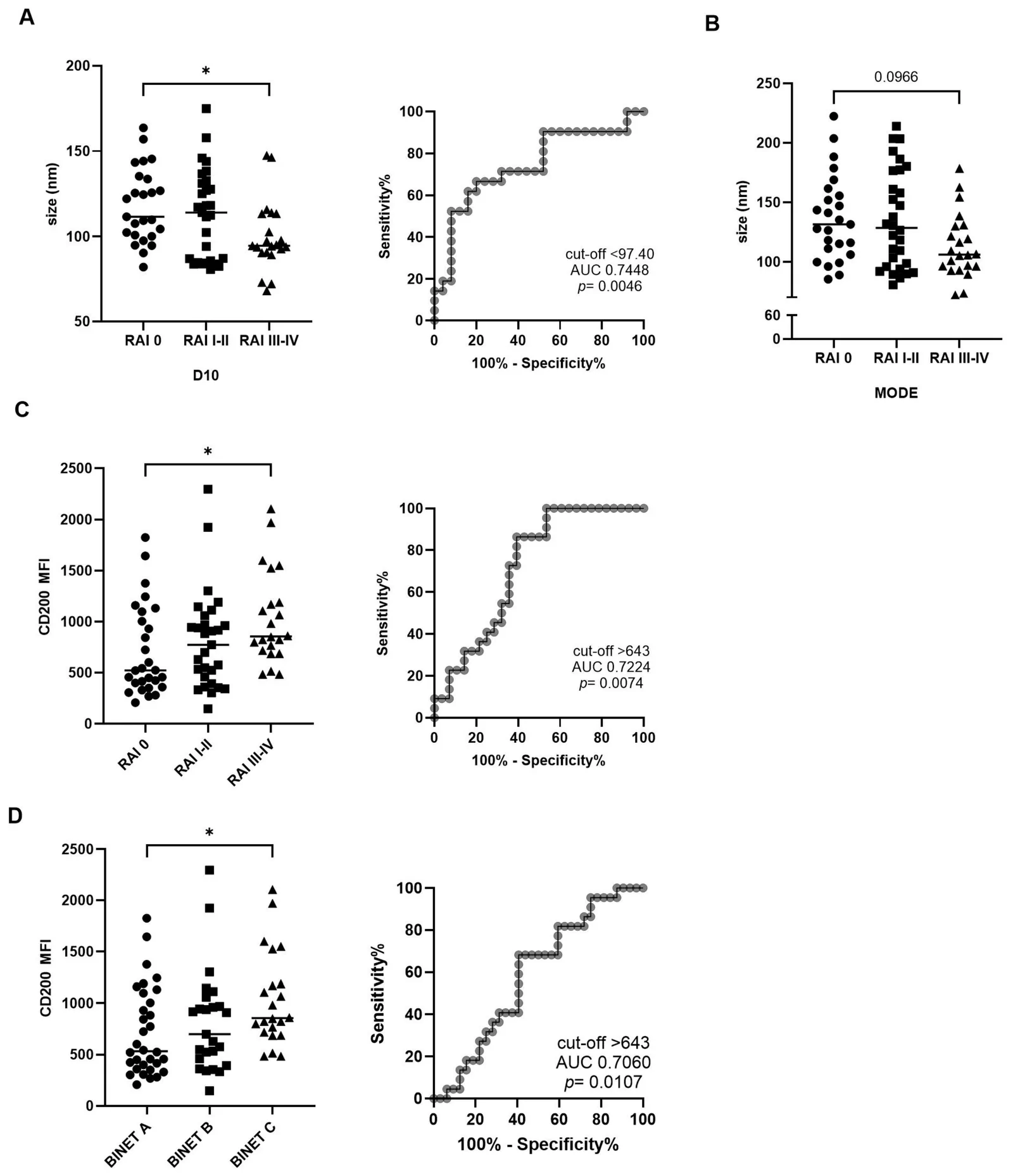
EV parameters and Rai/Binet stage of CLL patients. Dot plot and ROC curve comparisons of D10 (size, nm) (**A**). (**B**) Distribution of mode (size, nm) with Rai stage. (**C**) Dot plot and ROC curve comparison of CD200 MFI in CLL patients with different Rai stages. (**D**) Dot plot and ROC curve comparison of CD200 MFI in CLL patients with different Binet stages. Each symbol represents a single patient; statistically significant analyses are indicated by asterisks: * *p* < 0.05. Horizontal bars represent median values.

Subsequent analysis according to Rai stage (0, I–II, and III–IV) revealed significant differences in EV size and CD200 expression on EVs (Figure 7A–C). Specifically, D10 values were significantly lower in Rai stage III-IV than in Rai stage 0 patients (median, 94.60 vs. 111.5 nm, *p* < 0.05; Figure 7A). ROC curve analysis identified an optimal cut-off value of <97.4 nm (AUC 0.7448, 61.90% sensitivity and 84% specificity, *p* = 0.0046; Figure 7A). Similarly, the mode value tended to be lower in Rai stage III-IV than in Rai stage 0 patients (median, 106.1 vs. 131.4 nm, *p* = 0.0966; Figure 7B). In addition, CD200 MFI was significantly higher in Rai III-IV patients compared to Rai 0 patients (median, 855.4 vs. 523.1, *p* < 0.05; Figure 7C). ROC curve analysis identified an optimal cut-off value of >643 (AUC 0.7224, 86.36% sensitivity and 60.71% specificity, *p* = 0.0074; Figure 7C).

When patients were stratified according to the Binet staging system, the CD20^+^ EVs/µL value showed a trend toward lower values in Binet stage C compared with Binet stage A (median, 7988 vs. 9747 EVs/µL; *p* = 0.0924; Figure 7D). Conversely, CD200 MFI was significantly higher in Binet stage C than in Binet stage A patients (median, 855.4 vs. 533.7; *p* < 0.05; Figure 7D. ROC curve analysis identified a cut-off value of >643 (AUC 0.7060, 86.36% sensitivity, and 56.25% specificity, *p* = 0.0107; Figure 7D).

No significant differences were observed among Rai or Binet stage groups for the remaining EV parameters (Figures S6 and S7).

Regarding the FISH cytogenetic profile, CLL patients were stratified into favorable risk (patients with normal karyotype or del(13q)) and unfavorable risk (patients with trisomy 12, del(17p), del(11q), or two or more cytogenetic abnormalities). No significant differences in EV characteristics were observed between these two groups (Figure S8).

### 3.7. CD200^+^ EVs and CD19 Expression Showed Potential Predictive Value for Treatment Requirement and TTT in CLL Patients

To understand if EVs analyzed in the serum of CLL patients at diagnosis could predict whether the patients would require treatment, all patients were retrospectively stratified into two groups: patients who required treatment during follow-up (treated group, n = 27) and patients who remained untreated during a follow-up period of up to 200 months (untreated group, n = 55). One patient was excluded from the analysis because the received treatment was not for CLL.

**Figure 8:**
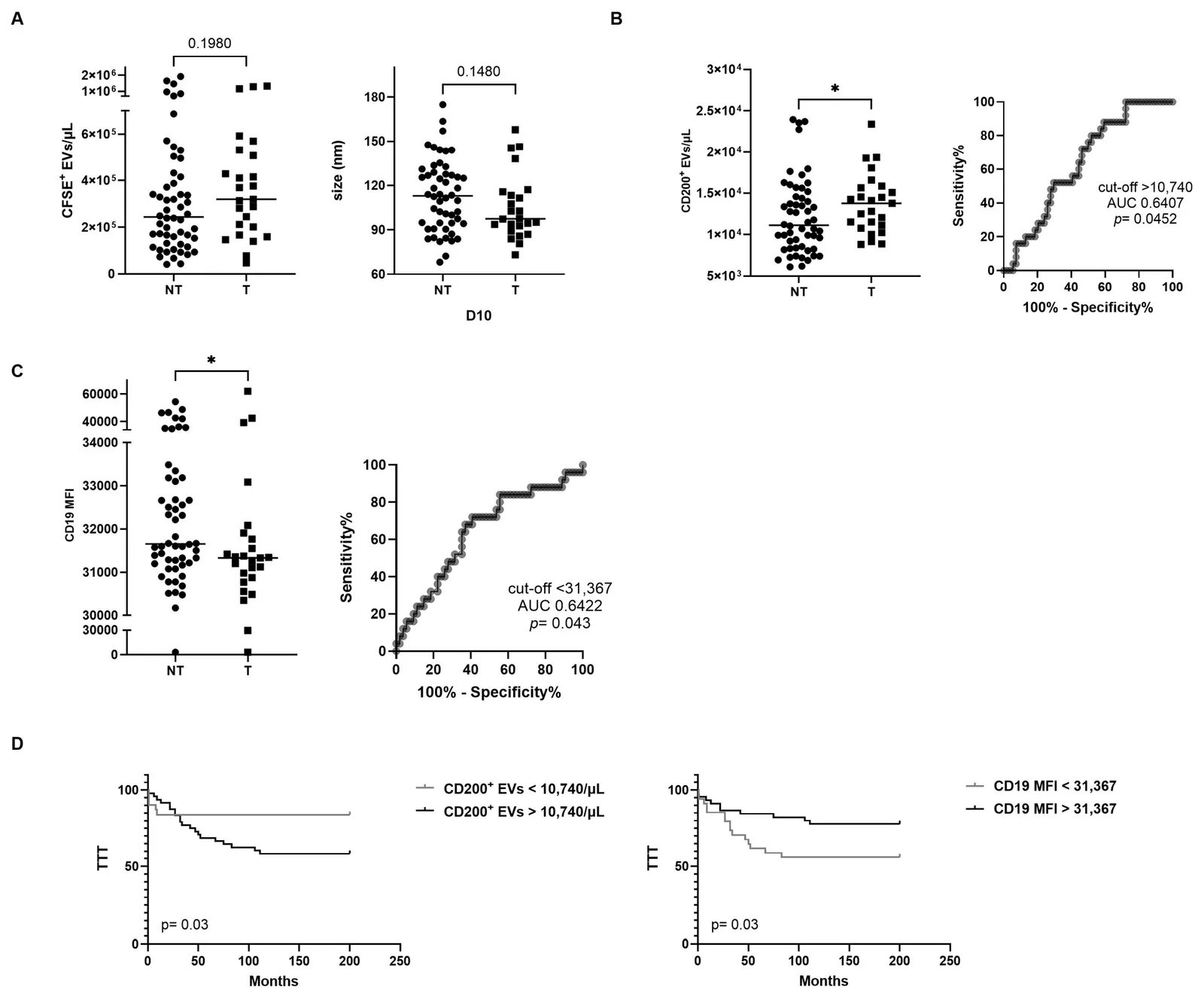
CLL EV predictive role for treatment or not for patients. (**A**) Dot plot of EV concentration (CFSE^+^ EVs/µL) and D10 (size, nm) comparison between CLL patients who will be treated (T) versus patients who will not be treated (NT). Dot plot and ROC curve comparison of (**B**) CD200^+^ EV/µL and (**C**) CD19 antigen expression level measured as MFI in NT or T CLL patients. Each symbol represents a single subject, and horizontal bars represent median values; statistically significant analyses are indicated by asterisks: * *p* < 0.05. (**D**) Kaplan–Meier curves obtained in CLL patients by comparing time to treatment (TTT) with higher number of CD200^+^ EVs (>10,740/µL; black line, left panel) or lower CD19 MFI (<31,367/µL; black line, right panel) with respect lower number of CD200^+^ EVs (<10,740/µL; gray line, left panel) or lower CD19 MFI (<31,367/µL; gray line, right panel). The log-rank (Mantel–Cox) test was used to compare two curves.

Patients who subsequently required treatment showed a trend toward higher EV concentration compared with those who remained untreated (median, 3.19 × 10^5^ vs. 2.44 × 10^5^ CFSE^+^ EVs/µL, *p* = 0.198; Figure 8A). In addition, the D10 parameter tended to be lower in treated patients than in untreated ones (median, 97.50 vs. 113 nm, *p* = 0.148; Figure 8A).

Notably, already at diagnosis, patients who subsequently required treatment displayed a significantly higher level of CD200^+^ EVs/µL compared with patients who remained untreated (median, 13,773 vs. 11,124 CD200^+^ EVs/µL, respectively, *p* = 0.0453; Figure 8B). ROC curve analysis established a cut-off value of >10,740 for predicting treatment requirement (AUC 0.6407, 48.15% sensitivity, and 80% specificity 80%, *p* = 0.045; Figure 8B).

Furthermore, CD19 MFI on EVs was significantly lower in patients who subsequently received treatment compared with those who remained untreated (median, 31,332 vs. 31,656, respectively, *p* = 0.0427; Figure 8C). ROC curve analysis identified a CD19 MFI cut-off value of >31,367 (AUC 0.6422, 64.81% sensitivity and specificity 60%, *p* = 0.043; Figure 8C). The ROC-derived cut-off values reported in this analysis should be considered exploratory, as they were generated and evaluated within the same patient cohort. Independent validation in larger, external cohorts will be required to confirm their predictive value.

To assess whether the combination of these biomarkers improved predictive performance, a multiple logistic regression model including the expression of CD200^+^ and CD19 MFI was generated. Although both CD200^+^ expression and CD19 MFI showed differential distribution between patients who subsequently required treatment and those who remained untreated, their combined analysis only marginally improved discrimination performance (AUC 0.64, *p* = 0.042), suggesting no additional predictive value of combining the two biomarkers.

Other EV-related parameters did not discriminate between these two groups of CLL patients (Figure S9).

As shown in Figure 8D, Kaplan–Meier analysis showed that patients with lower CD200^+^ EV levels had significantly longer TTT compared with patients with higher CD200^+^ EV levels (*p* = 0.03). Conversely, patients with lower CD19 MFI showed shorter TTT compared with patients with higher CD19 MFI (*p* = 0.03). These findings indicate an association between EV-associated biomarkers and TTT; however, their independent prognostic value requires validation in multivariate models including established clinical prognostic factors.

## 4. Discussion

The present study offers a comprehensive characterization of circulating EVs in CLL patients and HS and explores their potential diagnostic and prognostic significance in CLL.

We first demonstrated that CLL patients displayed an increased number of EVs, predominantly small and consistent with the B cell origin of CLL, and more interestingly, they also displayed CD200. Of note, CLL-derived EVs exhibit higher surface expression of CD20 and CD200 molecules, while maintaining the same CD19 levels when compared with their healthy counterpart. This pattern is different from the canonical immunophenotype of CLL cells, which typically display dim CD20 expression and up-regulated CD200 expression compared to normal B cells [3]. We hypothesized that in CLL, the presence of CD20 and CD200 on EVs may be more functionally relevant than their expression on the cell surface, potentially allowing broader systemic distribution and enhanced interaction with other cellular targets. In CLL, CD20 is involved in the activation, proliferation, and differentiation of B cells and interacts with the B cell receptor complex, thereby contributing to B cell activation and antibody production [20]. The presence of circulating CD20^+^ EVs may enhance the systemic effect of CLL on the entire body. As Martinez et al. reported in Non-Hodgkin Lymphoma [21], CLL CD20^+^ EVs may also interact with rituximab, interfering with therapy and contributing to immune escape, tumor progression, and potentially promoting therapeutic resistance within the tumor microenvironment.

Regarding CD200, to our knowledge, this is the first study reporting its expression on EVs in CLL. Few other studies reported that the interaction between CD200 and its receptor (CD200R) promotes immune evasion and tumor progression by suppressing immune responses and inhibiting tumor-specific T cell activity [22,23]. In a different context, breast tumor-derived EVs expressing immune regulatory molecules, including CD200, contributed to immune suppression through interaction with CD200R on immune cells [12]. We hypothesized that, similarly, CLL may exploit circulating CD200^+^ EVs to enhance its immunosuppressive effects. Consistently, in our cohort, CD200 expression appears to be greater on CLL-EVs compared with EVs from HS, mirroring the phenotype observed in CLL cells. Functional studies are required to demonstrate CD200^+^ EV biological role in CLL.

At the molecular level, we investigated the expression profile of four microRNAs in serum-derived EVs, specifically miR-93-5p, miR-125b-5p, miR-150-5p, and miR-484, based on two main considerations. First, these miRNAs were previously identified in other hematological malignancies, like acute myeloid leukemia and multiple myeloma, through a small RNA sequencing analyses combined with a systematic literature review. Second, increasing evidence indicates that these miRNAs play relevant roles in tumor initiation and progression, supporting the hypothesis that they may also contribute to the molecular mechanisms underlying CLL pathogenesis.

Although their functional role in CLL-derived EVs remains incompletely characterized, their detection in EVs supports their potential use as disease-associated biomarkers. miR-125b and miR-150 have been extensively characterized in CLL biology [14]. miR-125b has been reported to be associated with more aggressive disease [24], whereas miR-150 plays a role in B cell receptor (BCR) signaling pathways [14]. Conversely, the involvement of miR-484 and miR-93-5p in CLL remains unclear. Reduced miR-484 expression has been described in CLL subsets characterized by immune dysregulation, suggesting a potential role in leukemic cell–immune system interactions [25]. Beyond CLL, miR-484 is involved in solid and hematological malignancies, where it modulates cell proliferation, apoptosis, mitochondrial function, inflammation, and tumor progression. Notably, miR-484 displayed either tumor-suppressive or oncogenic properties depending on the cellular environment and disease setting [26]. In this context, the reduced levels of EV miR-484 in our CLL cohort may reflect disease-related alterations in miRNA regulation and EV biology, supporting the hypothesis that miR-484 could contribute to CLL-associated mechanisms. Further functional studies will be required to clarify its specific role in CLL pathogenesis and its potential relevance as a disease biomarker or therapeutic target. In contrast, miR-93 has been reported as up-regulated in plasma samples from CLL patients and associated with the miR-106b∼25 cluster, which has been implicated in promoting proliferative and survival signaling pathways [27]. In the EV context, recent studies have identified EV-associated miR-150-5p both in CLL primary cells and patient plasma [11,28], whereas the presence of the other miRNAs investigated in CLL-derived EVs has not yet been reported.

To our knowledge, this is the first study to report the detection of miR-93-5p, miR-125b-5p, and miR-484 in CLL-derived EVs. Specifically, these miRNAs were significantly down-regulated in our CLL-EVs compared with HS.

It is well known that CLL EVs are internalized by target cells in the microenvironment, including monocytes, fibrocytes, and lymphocytes, thereby modulating their phenotypes and fate [11,28]. In this study, we reported that the selected EV-miRNAs are involved in the regulation of key signaling pathways, including TP53, JAK/STAT, and HIF activation. These pathways regulate proliferation and programmed cell death, commonly dysregulated in tumors [29,30,31].

Overall, this study confirms and extends our previous findings on CLL-derived EVs [9] by applying our optimized EV isolation protocol in combination with multiparametric EV analysis [17,18]. In the future, this approach could enable a comprehensive characterization of circulating EVs, including their concentration, size, surface markers—distribution, expression of the B cell markers CD19 and CD20, the assessment of the novel marker CD200, and the profiling of their miRNA cargo [17,18] with high specificity and sensitivity.

Consistent with our previous findings [9], no association was observed between B cell-derived EVs and peripheral lymphocyte count, despite lymphocytosis being the earliest hallmark of CLL. This could suggest that EV levels do not simply reflect the number of leukemic cells in peripheral blood but may better represent the overall disease burden.

Interestingly, we also investigated the relationship between aging, EVs, and CLL. Aging is a major risk factor for cancer development and is associated with changes in the immune system and cellular homeostasis, processes that may influence EV biogenesis [32,33]. However, the effect of aging on EV concentration remains controversial [34].

In our study, EV concentration increased with age in HS, consistent with a recent report by Ráez-Meseguer et al. [35], but it decreased in CLL patients, suggesting that the disease may alter age-related EV dynamics. We also found an age-associated reduction in B cell-derived EVs (CD19, CD20, and CD200), together with lower CD20 and CD200 expression and reduced EV-associated miR-93-5p and miR-484 levels. These findings could reflect age-related changes in immune cell function and EV biology and could be in line with the generally less aggressive disease observed in older CLL patients [36,37]. Given that CLL predominantly occurs in older individuals, future studies, including larger cohorts of older patients, would, therefore, be valuable to determine the robustness of these markers across different age groups.

Little is known about the prognostic significance of CD200 in CLL. Our results showed that advanced disease stages, according to Rai and Binet classifications, were associated with distinct EV signatures, including reduced small EV fractions and increased CD200 expression on EVs, indicating their potential role in predicting disease progression. We and others previously reported that EV concentration and specific EV surface markers (CD19, CD20, and CD37) correlate with established clinical risk parameters in CLL, including higher Rai stage and unfavorable prognostic features [9,38]. To our knowledge, this is the first study reporting CD200 on EVs and its relationship with CLL stage. Of note, recently, it was reported that an elevated CD200 expression on cells is strongly linked to CLL progression, with a positive association with disease clinical stage, WBC count, and lymphocyte percentage [39]. Another study showed that baseline serum CD200 levels correlate with more aggressive clinical features and reduced survival, further supporting the relevance of CD200 beyond its diagnostic value [5].

Importantly, in our study, higher levels of CD200^+^ EVs and lower CD19 expression on EV surface were observed in patients at CLL diagnosis who subsequently required treatment compared with those who did not, and both features were associated with a shorter TTT. Consistently, previous studies have shown that lower CD200 expression on CLL cells and higher levels of soluble CD200 at diagnosis are associated with adverse clinical features, including shorter TTT and higher Rai risk stages [40,41]. Our findings are, therefore, biologically consistent with previous evidence, suggesting that CLL cells from patients with a more aggressive disease phenotype may release CD200 through both soluble and EV-associated forms, resulting in reduced CD200 expression on the cell surface. Further studies are required to validate this hypothesis and to clarify the potential role of CD200-c EVs in CLL progression and treatment response [41]. It is important to underline that our data support the potential role of EVs, their CD19 expression, and CD200 amount in the prediction of time to treatment in CLL. However, without internal validation, the proposed cut-offs should be considered exploratory and hypothesis-generating, and their prognostic value requires confirmation in independent, prospectively collected cohorts before clinical application.

The therapeutic landscape of CLL has rapidly evolved with the introduction of targeted therapies, including B cell receptor pathway inhibitors and BCL2 inhibitors, which have improved patient outcomes and shifted treatment approaches toward precision medicine [42]. However, CLL remains a heterogeneous disease with variable progression and treatment response, highlighting the need for novel biomarkers to refine risk stratification and guide clinical decisions. In this context, circulating EVs may represent a source of biological information, potentially complementing current prognostic tools and supporting personalized management of CLL patients. In line with this concept, and as observed in our previous study [9], we found higher levels and smaller size of circulating EVs in treated patients compared with untreated patients.

Our EV profiling, including the novel CD200, could have future clinical implications in current CLL diagnostics. In particular, EV strategy (i) is technically feasible and cost-effective, as it does not require commercial isolation kits and relies on instrumentation commonly available in routine clinical laboratories. (ii) It could improve patient stratification by identifying molecular features associated with disease behavior; (iii) it may provide additional biological information for the management of patients with early-stage CLL, a clinical setting in which treatment decisions and risk assessment remain particularly challenging; (iv) it could give supplementary insights into tumor biology by characterizing the molecular cargo and phenotype of tumor-associated EVs; and (v) it could supply relevant information during targeted therapy, such as anti-CD20 or potential future anti-CD200 treatment (CD20^+^ and/or CD200^+^ EV could sequester therapeutic antibodies, potentially reducing their availability to target malignant cells and contributing to treatment resistance or lack of response) [43,44].

However, these potential applications require validation in larger, independent, and prospective cohorts before EV analysis can be considered for routine clinical implementation.

## 5. Conclusions

Overall, our findings reinforced the importance of emerging EV multiparameter analysis (size, amount, antigen profile, and miRNA content) in CLL diagnostics and added the emerging new role of CD200, not only in soluble form and on the cell surface but now also on EVs, as a potential novel biomarker in CLL, supporting its potential diagnostic, prognostic, and predictive relevance.

## 6. Patents

WO2025078947A1—method for detecting the presence of a pathology in a subject; the method is based on the analysis of extracellular vesicles—Google Patents.

## Figures and Tables

**Table 1:** Clinicopathological features of CLL patients.

Parameter		Patients (Total n = 83)	%
Gender	Male	51	61.44
	Female	32	38.55
Age (years)	41–50	6	7.2
	51–60	22	26.5
	61–70	23	27.7
	71–80	21	25.3
	>80	11	13.3
Leucocytes (10^9^/L)	Median (range)	17.83 (2.3–205.4)	-
Lymphocytes (10^9^/L)	Median (range)	19.55 (0.85–195.9)	-
Hemoglobin (g/dL)	Median (range)	13.25 (6.7–16.8)	-
Platelets (10^9^/L)	Median (range)	167 (5–426)	-
Rai stage	0	28	33.7
	I–II	33	39.8
	III–IV	22	26.5
Binet stage	A	32	38.6
	B	29	34.9
	C	22	26.5
FISH	Normal	27	32.5
	Del(13)(q14)	17	20.5
	Trisomy 12	6	7.2
	Del(17)	2	2.4
	Del(11)(q22)	6	7.2
	Del(17) and del(13)(q14)	1	1.2
	Del(17) and del(11)(q22)	1	1.2
	Trisomy 12 and del(13)(q14)	1	1.2
	Not tested	22	26.5
Treatment	Yes	27	32.5
	No	55	66.3
	Tot for CLL	1	1.2
Status	Alive	45	54.2
	Dead	18	21.7
	Not available	20	24.1

## Data Availability

Data are available from the corresponding authors upon reasonable request.

## Supplementary Materials

The following supporting information can be downloaded at: https://www.mdpi.com/article/10.3390/biom16091293/s1, Figure S1: Dot plots of flow cytometric phenotypic analysis of (A) CLL cell line, HG3, and (B) HG3-derived EVs. Both HG3 cells and CFSE-labeled EVs were stained for CD19 APC, CD20 PE, CD200 PE, and their respective isotype (IgG1k PE or APC); Figure S2: Dot plots of D10 and mode in EVs from healthy subjects compared to EVs from CLL patients. Each symbol represents a single subject, and horizontal bars represent median values with 95% CI; Figure S3: Dot plots of CD19 MFI in EVs from healthy subjects compared to EVs from CLL patients. Each symbol represents a single sample, and horizontal bars represent median values with 95% CI; Figure S4: Dot plot of EV miR-150-5p level comparison between healthy subjects and CLL. Each symbol represents a single sample, and horizontal bars represent median values; Figure S5: Dot plot correlations between (A) CLL patient age and size (D10, D90, mean, and mode), CD19^+^/CD20^+^ EVs/µL, CD19 MFI, EV miR-125b-5p, and EV miR-150-5p expression. R and p are indicated on plots. (B) Healthy subject age size (D10, D90, mean, and mode), CD19^+^/CD20^+^ EVs/µL, CD20 MFI, CD19 MFI, EV miR-125b-5p, EV and miR-150-5p, expression; Figure S6: Plots of the trend of EV parameters (concentration by NTA and cytometer, D90, mean, CD20^+^ EVs/µL, CD19^+^ EVs/µL, CD200^+^ EVs/µL, CD19^+^/CD20^+^ EVs/µL, CD20 MFI, CD19 MFI, EV miR-125b-5p, EV miR-93, EV miR-484, and EV miR-150-5p amount (copies/µL) in CLL patients according to Rai stage; Figure S7: Plots of the trend of EV parameters (concentration by NTA and cytometer, D10, D90, mean, mode, CD19^+^ EVs/µL, CD200^+^ EVs/µL, CD19^+^/CD20^+^ EVs/µL, CD20 MFI, CD19 MFI, EV miR-93, EV miR-125b-5p, EV miR-484, and EV miR-150-5p amount (copies/µL) in CLL patients according to BINET stage; Figure S8: Dot plot of EV parameter (concentration by NTA and cytometer, D10, D90, mean, mode, CD19^+^ EVs/µL, CD20^+^ EVs/µL, CD19^+^/CD20^+^ EVs/µL, CD200^+^ EVs/µL, CD19 MFI, CD20 MFI, and CD200 MFI EV) comparison between CLL patients with favorable FISH and unfavorable ones. Each symbol represents a single sample, and horizontal bars represent median values. Statistical analyses are reported on plots; Figure S9: Dot plot of EV parameter comparison (concentration by NTA, D90, mean, mode, CD19^+^ EVs/µL, CD20^+^ EVs/µL, CD19^+^/CD20^+^ EVs/µL, CD20 MFI, and CD200 MFI EV, EV miR-93, EV miR-125b-5p, EV miR-484, and EV miR-150-5p amount (copies/µL)) between CLL patients who will be treated (YES) versus patients who will not be treated (NO). Each symbol represents a single sample, and horizontal bars represent median values. Statistical analyses are reported on plots; Table S1. Clinicopathological features of 20 CLL patients included in the EV miRNA analyses. Table S2: miRNA RNA targets.

## Abbreviations

The following abbreviations are used in this manuscript:

APC	Allophycocyanin
AUC	Area Under the Curve
CFDA-SE	5-Carboxyfluorescein Diacetate-Succinimidyl Ester
CFSE	Carboxyfluoresceinsuccinimidyl Ester
CLL	Chronic Lymphocytic Leukemia
ddPCR	Droplet Digital PCR
EVs	Extracellular Vesicles
HS	Healthy Subjects
IGHV	Immunoglobulin Heavy Chain Variable region
MFI	Mean Fluorescence Intensity
miRNAs, miR	microRNAs
NTA	Nanoparticle Tracking Analysis
PB	Peripheral Blood
PBS	Phosphate-Buffered Saline
PE	Phycoerythrin
ROC	Receiver Operating Characteristic
sCD200	Soluble Ectodomain CD200
TEM	Transmission Electron Microscope
TTT	Time To Treatment
