# Supplementary material for: Circulating Extracellular Vesicle Biomarkers in Chronic Lymphocytic Leukemia: A Preliminary Study on Their Diagnostic, Prognostic, and Predictive Relevance
Source: Biomolecules. 2026 Sep 7;16(9):1293. doi: 10.3390/biom16091293 (PMC13604536; doi:10.3390/biom16091293)
Supplement: Supplementary file 1 [file biomolecules-16-01293-s001.zip › biomolecules-4496725-supplementary.pdf]

Supplementary Materials

Table S1. Clinicopathological features of 20 CLL patients included in the EV miRNA analyses.

**Parameter**		**Patients (total = 20)**	**%**
**Gender**	Male	14	70
	Female	6	30
**Age (years)**	51-60	3	15
	61-70	9	45
	71-80	6	30
	>80	2	10
**Rai stage**	0	5	25
	I-II	5	25
	III-IV	10	50
**BINET**	A	5	25
	B	5	25
	C	10	50
**FISH**	normal	5	25
	del(139(q14)	6	30
	del(17)	1	5
	del(11)(q22)	2	10
	Not tested	6	30
**Treatment**	yes	6	30
	no	14	70
**Status**	alive	9	45
	dead	5	25
	Not available	6	30

Table S2. miRNA RNA targets.

miRTargetLink 2.0					
**miRNA**	**Target / Target pathway**	**Support**	**Source**	**Experiment(s)**	**Reference - PMID**
hsa-miR-125b-5p	SEMA4C	Functional MTI	MIRT501239	Luciferase reporter assay, qRT-PCR, Western blot	25605244
hsa-miR-125b-5p	SPHK1	Functional MTI	MIRT731325	Luciferase reporter assay	26807182
hsa-miR-125b-5p	MMP2	Functional MTI	MIRT731453	Luciferase reporter assay	27133077
hsa-miR-125b-5p	MMP26	Functional MTI	MIRT731486	Luciferase reporter assay	27143441
hsa-miR-125b-5p	SFRP5	Functional MTI	MIRT731695	Luciferase reporter assay	27592695
hsa-miR-125b-5p	MAP3K11	Functional MTI	MIRT731595	Luciferase reporter assay, Microarray, qRT-PCR, Western blot	26138442
hsa-miR-125b-5p	MAP3K11	Functional MTI	MIRT731595	Luciferase reporter assay, qRT-PCR, Western blot	27613090
hsa-miR-125b-5p	GAB2	Functional MTI	MIRT732555	Luciferase reporter assay, qRT-PCR, Western blot	26354435
hsa-miR-125b-5p	LIFR	Functional MTI	MIRT732494	Microarray, Western blot	25915540
hsa-miR-125b-5p	IGF1R	Functional MTI	MIRT733120	Luciferase reporter assay, qRT-PCR	26455324
hsa-miR-125b-5p	Fas	Functional MTI	MIRT732691	ChIP-seq, Luciferase reporter assay, Microarray, qRT-PCR, Western blot	26272872
hsa-miR-125b-5p	APLN	Functional MTI	MIRT732889	Luciferase reporter assay, Next Generation Sequencing (NGS), qRT-PCR	26585673
hsa-miR-125b-5p	PIK3CB	Functional MTI	MIRT732929	qRT-PCR, Western blot	26458815
hsa-miR-125b-5p	CD44	Functional MTI	MIRT733342	Immunohistochemistry, Western blot	26686386
hsa-miR-125b-5p	NEU1	Functional MTI	MIRT733169	Immunohistochemistry, Luciferase reporter assay, qRT-PCR, Western blot	27220320
hsa-miR-125b-5p	ALOX5	Functional MTI	MIRT733210	Luciferase reporter assay, qRT-PCR, Western blot	25589070
hsa-miR-125b-5p	EGFR	Functional MTI	MIRT733343	Immunohistochemistry, Western blot	26686386
hsa-miR-125b-5p	TNF	Functional MTI	MIRT733472	ChIP, Luciferase reporter assay, qRT-PCR, Western blot	26435691
hsa-miR-125b-5p	FES	Functional MTI	MIRT734255	Luciferase reporter assay, Western blot	28389358
hsa-miR-125b-5p	PHF8	Functional MTI	MIRT733632	Luciferase reporter assay	26309412
hsa-miR-125b-5p	ANGPT2	Functional MTI	MIRT733867	Immunoblot, Luciferase reporter assay	27577856
hsa-miR-125b-5p	APC	Functional MTI	MIRT734087	Luciferase reporter assay, qRT-PCR, Western blot	28176874
hsa-miR-125b-5p	KLC2	Functional MTI	MIRT733658	Luciferase reporter assay, qRT-PCR, Western blot	25668010
hsa-miR-125b-5p	DKK3	Functional MTI	MIRT734683	ChIP-seq, Luciferase reporter assay, qRT-PCR, Western blot	27788486
hsa-miR-125b-5p	FZD6	Functional MTI	MIRT734714	Luciferase reporter assay	27698350
hsa-miR-125b-5p	CDKN2D	Functional MTI	MIRT734885	Luciferase reporter assay, qRT-PCR, Western blot	27763644
hsa-miR-125b-5p	SUV39H1	Functional MTI	MIRT735058	qRT-PCR, Western blot	27304919
hsa-miR-125b-5p	PTH1R	Functional MTI	MIRT735236	Luciferase reporter assay, qRT-PCR, Western blot	26351562
hsa-miR-125b-5p	PODXL	Functional MTI	MIRT734954	Luciferase reporter assay	25738314
hsa-miR-125b-5p	JAK2	Functional MTI	MIRT734987	qRT-PCR, Western blot	26011312
hsa-miR-125b-5p	HOTTIP	Functional MTI	MIRT735307	Luciferase reporter assay, qRT-PCR	25424744
hsa-miR-125b-5p	DRAM2	Functional MTI	MIRT735480	Luciferase reporter assay, qRT-PCR, Western blot	27518550

hsa-miR-125b-5p	NKIRAS2	Functional MTI	MIRT000462	ELISA, qRT-PCR, Luciferase reporter assay, Western blot	20351193
hsa-miR-125b-5p	BMPR1B	Functional MTI	MIRT000346	Luciferase reporter assay	19738052
hsa-miR-125b-5p	BMPR1B	Functional MTI	MIRT000346	Luciferase reporter assay, qRT-PCR, Western blot	28214897
hsa-miR-125b-5p	EIF4EBP1	Functional MTI	MIRT000347	Immunoblot, Immunohistochemistry, In situ hybridization, Luciferase reporter assay, Micro-array, qRT-PCR	22071331
hsa-miR-125b-5p	EIF4EBP1	Functional MTI	MIRT000347	Luciferase reporter assay, qRT-PCR, Western blot	26646586
hsa-miR-125b-5p	HMGA2	Functional MTI	MIRT000348	Luciferase reporter assay	17563749
hsa-miR-125b-5p	HMGA1	Functional MTI	MIRT000349	Luciferase reporter assay	17563749
hsa-miR-125b-5p	GLI1	Functional MTI	MIRT000350	Luciferase reporter assay	18756266
hsa-miR-125b-5p	TP53	Functional MTI	MIRT000535	Next Generation Sequencing (NGS), qRT-PCR, Western blot	26585673
hsa-miR-125b-5p	SMO	Functional MTI	MIRT000525	Luciferase reporter assay, Western blot	20216554
hsa-miR-125b-5p	SMO	Functional MTI	MIRT000525	Luciferase reporter assay, Western blot	18756266
hsa-miR-125b-5p	TP53	Functional MTI	MIRT000535	Luciferase reporter assay, Western blot	20216554
hsa-miR-125b-5p	TP53	Functional MTI	MIRT000535	In situ hybridization, Luciferase reporter assay, qRT-PCR, Western blot, Reporter assay;qRT-PCR;Other	19293287
hsa-miR-125b-5p	TP53	Functional MTI	MIRT000535	Luciferase reporter assay, qRT-PCR, Western blot	21935352
hsa-miR-125b-5p	TP53	Functional MTI	MIRT000535	Luciferase reporter assay	23497288
hsa-miR-125b-5p	TP53	Functional MTI	MIRT000535	Luciferase reporter assay, Western blot	22331826
hsa-miR-125b-5p	VDR	Functional MTI	MIRT001197	Luciferase reporter assay, Western blot	19437538
hsa-miR-125b-5p	VDR	Functional MTI	MIRT001197	qRT-PCR, Western blot	22213330
hsa-miR-125b-5p	ERBB3	Functional MTI	MIRT002938	Luciferase reporter assay, Northern blot, qRT-PCR, Western blot	17110380
hsa-miR-125b-5p	SGPL1	Functional MTI	MIRT002370	Luciferase reporter assay	19635812
hsa-miR-125b-5p	BAK1	Functional MTI	MIRT002394	Luciferase reporter assay, qRT-PCR, Western blot	18056640
hsa-miR-125b-5p	BAK1	Functional MTI	MIRT002394	Luciferase reporter assay, qRT-PCR, Western blot	21823019
hsa-miR-125b-5p	BAK1	Functional MTI	MIRT002394	Luciferase reporter assay	23497288
hsa-miR-125b-5p	BAK1	Functional MTI	MIRT002394	Luciferase reporter assay,	23928699
hsa-miR-125b-5p	BAK1	Functional MTI	MIRT002394	Luciferase reporter assay	27158338
hsa-miR-125b-5p	ERBB3	Functional MTI	MIRT002938	Western blot, qRT-PCR	23519125
hsa-miR-125b-5p	ERBB2	Functional MTI	MIRT002939	Western blot	19825990
hsa-miR-125b-5p	ERBB2	Functional MTI	MIRT002939	Luciferase reporter assay, Northern blot, qRT-PCR, Western blot	17110380
hsa-miR-125b-5p	ERBB2	Functional MTI	MIRT002939	Luciferase reporter assay, Microarray, Western blot	20864407
hsa-miR-125b-5p	ERBB2	Functional MTI	MIRT002939	Luciferase reporter assay, Western blot	22460089
hsa-miR-125b-5p	ERBB2	Functional MTI	MIRT002939	qRT-PCR, Luciferase reporter assay, Immunoblot, Western blot	25388283
hsa-miR-125b-5p	ERBB2	Functional MTI	MIRT002939	Luciferase reporter assay, Western blot	26966351
hsa-miR-125b-5p	ERBB2	Functional MTI	MIRT002939	Luciferase reporter assay	26544868
hsa-miR-125b-5p	ERBB2	Functional MTI	MIRT002939	Luciferase reporter assay, qRT-PCR, Western blot	25833836
hsa-miR-125b-5p	ERBB3	Functional MTI	MIRT002938	Immunoblot, Immunohistochemistry, Luciferase reporter assay, qRT-PCR	23146892

hsa-miR-125b-5p	BMF	Functional MTI	MIRT003394	immunoblot, Luciferase reporter assay, qRT-PCR	19471102
hsa-miR-125b-5p	KLF13	Functional MTI	MIRT003421	Microarray, qRT-PCR, Western blot	19396866
hsa-miR-125b-5p	NTRK3	Functional MTI	MIRT003754	Luciferase reporter assay, Western blot, Reporter assay	17483472
hsa-miR-125b-5p	CBFB	Functional MTI	MIRT003970	Luciferase reporter assay, qRT-PCR, Western blot	21903586
hsa-miR-125b-5p	CBFB	Functional MTI	MIRT003970	Luciferase reporter assay	22689670
hsa-miR-125b-5p	LIN28A	Functional MTI	MIRT003845	Reporter assay	18230348
hsa-miR-125b-5p	LIN28A	Functional MTI	MIRT003845	B-globin reporter assay, Luciferase reporter assay	16495412
hsa-miR-125b-5p	LIN28A	Functional MTI	MIRT003845	Luciferase reporter assay	16227573
hsa-miR-125b-5p	LIN28A	Functional MTI	MIRT003845	Immunoblot, Luciferase reporter assay, qRT-PCR	22366319
hsa-miR-125b-5p	LIN28A	Functional MTI	MIRT003845	Luciferase reporter assay, Western blot	22277001
hsa-miR-125b-5p	AKT1	Functional MTI	MIRT004363	qRT-PCR, Western blot	18649363
hsa-miR-125b-5p	RAF1	Functional MTI	MIRT004418	Western blot, Western blot;Other	19825990
hsa-miR-125b-5p	CYP24A1	Functional MTI	MIRT004408	qRT-PCR, Luciferase reporter assay, Western blot, Northern blot, Reporter assay	19570947
hsa-miR-125b-5p	PRDM1	Functional MTI	MIRT004533	qRT-PCR, Luciferase reporter assay	20497960
hsa-miR-125b-5p	PRDM1	Functional MTI	MIRT004533	Chromatin immunoprecipitation, Immunoblot, Luciferase reporter assay, Microarray, qRT-PCR	26655851
hsa-miR-125b-5p	PRDM1	Functional MTI	MIRT004533	Luciferase reporter assay, qRT-PCR, Western blot	27613090
hsa-miR-125b-5p	IRF4	Functional MTI	MIRT004534	qRT-PCR, Luciferase reporter assay	20497960
hsa-miR-125b-5p	GRIN2A	Functional MTI	MIRT004578	Luciferase reporter assay, qRT-PCR	20159450
hsa-miR-125b-5p	CGN	Functional MTI	MIRT004994	Western blot	28082316
hsa-miR-125b-5p	LACTB	Functional MTI	MIRT004998	Luciferase reporter assay, Western blot	26603571
hsa-miR-125b-5p	STAT3	Functional MTI	MIRT005006	Western blot	28108732
hsa-miR-125b-5p	STAT3	Functional MTI	MIRT005006	Luciferase reporter assay, Western blot	27576314
hsa-miR-125b-5p	CDKN2A	Functional MTI	MIRT004709	Western blot	20347935
hsa-miR-125b-5p	CDKN2A	Functional MTI	MIRT004709	Immunoprecipitation, Luciferase reporter assay, Western blot	23585871
hsa-miR-125b-5p	TP53INP1	Functional MTI	MIRT004996	Luciferase reporter assay, Northern blot, qRT-PCR, Western blot	21970405
hsa-miR-125b-5p	TP53INP1	Functional MTI	MIRT004996	qRT-PCR, Western blot	26388699
hsa-miR-125b-5p	E2F3	Functional MTI	MIRT005503	Immunohistochemistry, Luciferase reporter assay, Northern blot, qRT-PCR, Western blot, Reporter assay;Other	20549700
hsa-miR-125b-5p	E2F3	Functional MTI	MIRT005503	Immunohistochemistry, qRT-PCR, Western blot	22523546
hsa-miR-125b-5p	IGF2	Functional MTI	MIRT005738	Luciferase reporter assay, Northern blot, Western blot	21200031
hsa-miR-125b-5p	LIN28B	Functional MTI	MIRT005804	Luciferase reporter assay, qRT-PCR, Western blot	20827722
hsa-miR-125b-5p	BBC3	Functional MTI	MIRT005915	Luciferase reporter assay, qRT-PCR, Western blot	20886540
hsa-miR-125b-5p	PPP1CA	Functional MTI	MIRT006127	Luciferase reporter assay, qRT-PCR, Western blot	21935352
hsa-miR-125b-5p	PPP1CA	Functional MTI	MIRT006127	Immunohistochemistry, In situ hybridization, Luciferase reporter assay, Microarray, qRT-PCR, Western blot	25240408
hsa-miR-125b-5p	PPP1CA	Functional MTI	MIRT006127	Luciferase reporter assay, Western blot, qRT-PCR	25001178
hsa-miR-125b-5p	PRKRA	Functional MTI	MIRT006128	Luciferase reporter assay, qRT-PCR, Western blot	21935352

hsa-miR-125b-5p	BCL2	Functional MTI	MIRT006253	Luciferase reporter assay, Western blot	22293115
hsa-miR-125b-5p	ETS1	Functional MTI	MIRT006387	Immunoblot, Luciferase reporter assay	21444677
hsa-miR-125b-5p	RPS6KA1	Functional MTI	MIRT006430	Luciferase reporter assay, ELISA, GFP reporter assay, Immunoblot, Immunoprecipitaion, qRT-PCR, Western blot	21643012
hsa-miR-125b-5p	RPS6KA1	Functional MTI	MIRT006430	Luciferase reporter assay, qRT-PCR, Western blot	27613090
hsa-miR-125b-5p	ETS1	Functional MTI	MIRT006387	Western blot, qRT-PCR	24486760
hsa-miR-125b-5p	TBC1D1	Functional MTI	MIRT006523	Luciferase reporter assay	19635812
hsa-miR-125b-5p	DGAT1	Functional MTI	MIRT006524	Luciferase reporter assay	19635812
hsa-miR-125b-5p	TNFAIP3	Functional MTI	MIRT006472	qRT-PCR, Western blot	22550173
hsa-miR-125b-5p	TNFAIP3	Functional MTI	MIRT006472	Luciferase reporter assay, qRT-PCR, Western blot	27637078
hsa-miR-125b-5p	PIGF	Functional MTI	MIRT006495	Luciferase reporter assay, qRT-PCR, Western blot	21703189
hsa-miR-125b-5p	BCL3	Functional MTI	MIRT006500	Luciferase reporter assay	20658525
hsa-miR-125b-5p	FGFR2	Functional MTI	MIRT006678	Immunohistochemistry, Luciferase reporter assay, qRT-PCR, Western blot	21412257
hsa-miR-125b-5p	ARID3B	Functional MTI	MIRT006719	Luciferase reporter assay	22307404
hsa-miR-125b-5p	MCL1	Functional MTI	MIRT006843	Luciferase reporter assay, qRT-PCR, Western blot	22942733
hsa-miR-125b-5p	SMAD4	Functional MTI	MIRT006810	Luciferase reporter assay, Western blot	22357933
hsa-miR-125b-5p	MCL1	Functional MTI	MIRT006843	Luciferase reporter assay, Western blot	22824797
hsa-miR-125b-5p	MCL1	Functional MTI	MIRT006843	In situ hybridization, Luciferase reporter assay, qRT-PCR, Western blot	26504803
hsa-miR-125b-5p	IL6R	Functional MTI	MIRT006844	Luciferase reporter assay, qRT-PCR, Western blot	22942733
hsa-miR-125b-5p	IL6R	Functional MTI	MIRT006844	Luciferase reporter assay, Western blot	22824797
hsa-miR-125b-5p	STARD13	Functional MTI	MIRT006849	Luciferase reporter assay	22693547
hsa-miR-125b-5p	ABTB1	Functional MTI	MIRT006910	Luciferase reporter assay	22689670
hsa-miR-125b-5p	STARD13	Functional MTI	MIRT006849	Immunohistochemistry, Luciferase reporter assay, qRT-PCR, Western blot	27220320
hsa-miR-125b-5p	HK2	Functional MTI	MIRT006945	Luciferase reporter assay, qRT-PCR	22593586
hsa-miR-125b-5p	E2F2	Functional MTI	MIRT006974	Luciferase reporter assay, qRT-PCR, Western blot	22999819
hsa-miR-125b-5p	MMP13	Functional MTI	MIRT006990	Luciferase reporter assay	22782903
hsa-miR-125b-5p	MAPK14	Functional MTI	MIRT007066	ChIP-seq, EMSA, Immunoblot, Luciferase reporter assay, qRT-PCR, Western blot	22854965
hsa-miR-125b-5p	MUC1	Functional MTI	MIRT022114	Reporter assay;Western blot	20729973
hsa-miR-125b-5p	EPO	Functional MTI	MIRT022112	Luciferase reporter assay	24165569
hsa-miR-125b-5p	CDH5	Functional MTI	MIRT052977	qRT-PCR, Western blot	22391569
hsa-miR-125b-5p	ARID3A	Functional MTI	MIRT052991	Luciferase reporter assay, qRT-PCR, Western blot	22469780
hsa-miR-125b-5p	NCOR2	Functional MTI	MIRT053093	Luciferase reporter assay, Western blot	23514806
hsa-miR-125b-5p	EIF5A2	Functional MTI	MIRT053126	Luciferase reporter assay, qRT-PCR, Western blot	24811246
hsa-miR-125b-5p	NES	Functional MTI	MIRT035540	Luciferase reporter assay	23016664
hsa-miR-125b-5p	CEBPA	Functional MTI	MIRT045932	Luciferase reporter assay, qRT-PCR, Western blot	25982911
hsa-miR-125b-5p	BCL2L2	Functional MTI	MIRT053019	Luciferase reporter assay, Western blot	22824797
hsa-miR-125b-5p	BCL2L2	Functional MTI	MIRT053019	Luciferase reporter assay, Western blot, qRT-PCR	25001178

hsa-miR-125b-5p	MXD1	Functional MTI	MIRT053210	Immunofluorescence, Immunohistochemistry, Luciferase reporter assay, qRT-PCR, Western blot	23099851
hsa-miR-125b-5p	PIAS3	Functional MTI	MIRT053294	Luciferase reporter assay, Western blot	23857508
hsa-miR-125b-5p	PIK3CD	Functional MTI	MIRT053622	Luciferase reporter assay, qRT-PCR, Western blot	23160634
hsa-miR-125b-5p	PIK3CD	Functional MTI	MIRT053622	Luciferase reporter assay, qRT-PCR, Western blot	24517182
hsa-miR-125b-5p	BTG2	Functional MTI	MIRT061544	Luciferase reporter assay, qRT-PCR, Western blot	27613090
hsa-miR-125b-5p	ICAM2	Functional MTI	MIRT054251	Luciferase reporter assay, QRTPCR	23591197
hsa-miR-125b-5p	VPS4B	Functional MTI	MIRT054863	Luciferase reporter assay	25403182
hsa-miR-125b-5p	PCTP	Functional MTI	MIRT054197	Luciferase reporter assay, Microarray, qRT-PCR, Western blot	22723551
hsa-miR-125b-5p	LIPA	Functional MTI	MIRT054198	Luciferase reporter assay, Microarray, qRT-PCR, Western blot	22723551
hsa-miR-125b-5p	GSS	Functional MTI	MIRT054199	Luciferase reporter assay, Microarray, qRT-PCR, Western blot	22723551
hsa-miR-125b-5p	IKZF2	Functional MTI	MIRT054200	Luciferase reporter assay, Microarray, qRT-PCR, Western blot	22723551
hsa-miR-125b-5p	IKZF3	Functional MTI	MIRT054201	Luciferase reporter assay, Microarray, qRT-PCR, Western blot	22723551
hsa-miR-125b-5p	IKZF4	Functional MTI	MIRT054202	Luciferase reporter assay, Microarray, qRT-PCR, Western blot	22723551
hsa-miR-125b-5p	ENPEP	Functional MTI	MIRT437377	Luciferase reporter assay, Western blot	24098452
hsa-miR-125b-5p	SET	Functional MTI	MIRT259021	Luciferase reporter assay, qRT-PCR, Western blot	27383536
hsa-miR-125b-5p	CCNJ	Functional MTI	MIRT263904	Luciferase reporter assay, Western blot	24098452
hsa-miR-125b-5p	CSNK2A1	Functional MTI	MIRT437426	Luciferase reporter assay, Western blot	24098452
hsa-miR-125b-5p	MEGF9	Functional MTI	MIRT437427	Luciferase reporter assay, Western blot	24098452
hsa-miR-125b-5p	AHRR	Functional MTI	MIRT438200	Immunoblot, Luciferase reporter assay	24176857
hsa-miR-125b-5p	MAN1B1	Functional MTI	MIRT438070	Luciferase reporter assay, qRT-PCR, Western blot	23940818
hsa-miR-125b-5p	EPOR	Functional MTI	MIRT438152	Luciferase reporter assay	24165569
hsa-miR-125b-5p	SCNN1A	Functional MTI	MIRT438382	Luciferase reporter assay, qRT-PCR, Western blot	25173609
hsa-miR-125b-5p	VPS51	Functional MTI	MIRT438383	Luciferase reporter assay	25172656
hsa-miR-125b-5p	DUSP6	Functional MTI	MIRT438607	Luciferase reporter assay, Western blot, qRT-PCR	25001178
hsa-miR-125b-5p	SIRT7	Functional MTI	MIRT438446	Flow, Immunoblot, Luciferase reporter assay, Microarray, Northern blot, qRT-PCR, Western blot	23079745
hsa-miR-125b-5p	SIRT7	Functional MTI	MIRT438446	Luciferase reporter assay, qRT-PCR, Western blot	26770454
hsa-miR-125b-5p	TET2	Functional MTI	MIRT438630	Luciferase reporter assay	24120864
hsa-miR-150-5p	SSSCA1	Functional MTI	MIRT731116	qRT-PCR, Luciferase reporter assay, Western blot	25435958
hsa-miR-150-5p	STAT5B	Functional MTI	MIRT732355	Luciferase reporter assay, qRT-PCR, Western blot	27447965
hsa-miR-150-5p	CREB1	Functional MTI	MIRT732237	Luciferase reporter assay, qRT-PCR, Western blot	27285761
hsa-miR-150-5p	STAT1	Functional MTI	MIRT733223	Luciferase reporter assay, qRT-PCR, Western blot	26025667
hsa-miR-150-5p	POLD3	Functional MTI	MIRT733573	Immunofluorescence, Luciferase reporter assay, qRT-PCR	27862371
hsa-miR-150-5p	NANOG	Functional MTI	MIRT733717	Immunohistochemistry, Luciferase reporter assay, qRT-PCR, Western blot	27917123
hsa-miR-150-5p	PRKCA	Functional MTI	MIRT734463	Luciferase reporter assay, qRT-PCR, Western blot	26232986
hsa-miR-150-5p	MMP14	Functional MTI	MIRT734650	Luciferase reporter assay, qRT-PCR, Western blot	25549355

hsa-miR-150-5p	ARRB2	Functional MTI	MIRT734904	Luciferase reporter assay, qRT-PCR, Western blot	26549736
hsa-miR-150-5p	SLC2A1	Functional MTI	MIRT735450	Luciferase reporter assay, Western blot	26746193
hsa-miR-150-5p	ZNF350	Functional MTI	MIRT735367	Luciferase reporter assay, qRT-PCR, Western blot	27586871
hsa-miR-150-5p	MYB	Functional MTI	MIRT000935	Western blot, Luciferase reporter assay, Reporter assay;Western blot;Other	18667440
hsa-miR-150-5p	MYB	Functional MTI	MIRT000935	Luciferase reporter assay, Western blot	18539114
hsa-miR-150-5p	MYB	Functional MTI	MIRT000935	Luciferase reporter assay, qRT-PCR, Western blot	18814950
hsa-miR-150-5p	MYB	Functional MTI	MIRT000935	Luciferase reporter assay	20603081
hsa-miR-150-5p	MYB	Functional MTI	MIRT000935	Reporter assay	17923094
hsa-miR-150-5p	MYB	Functional MTI	MIRT000935	Flow, Luciferase reporter assay, Microarray, qRT-PCR, Western blot	22025269
hsa-miR-150-5p	MYB	Functional MTI	MIRT000935	Luciferase reporter assay, Microarray, qRT-PCR	23604034
hsa-miR-150-5p	MYB	Functional MTI	MIRT000935	Immunohistochemistry, In situ hybridization, Microarray, qRT-PCR, Western blot	24613688
hsa-miR-150-5p	MYB	Functional MTI	MIRT000935	Luciferase reporter assay, qRT-PCR, Western blot	24438945
hsa-miR-150-5p	MYB	Functional MTI	MIRT000935	Luciferase reporter assay, Microarray	24086639
hsa-miR-150-5p	MYB	Functional MTI	MIRT000935	Flow, qRT-PCR, Western blot	23521217
hsa-miR-150-5p	EGR2	Functional MTI	MIRT003222	Luciferase reporter assay, semi-qRT-PCR, Western blot	20067763
hsa-miR-150-5p	EGR2	Functional MTI	MIRT003222	Luciferase reporter assay, Microarray, qRT-PCR	23604034
hsa-miR-150-5p	VEGFA	Functional MTI	MIRT004272	ELISA, Luciferase reporter assay	18320040
hsa-miR-150-5p	P2RX7	Functional MTI	MIRT004357	qRT-PCR, Luciferase reporter assay	18682393
hsa-miR-150-5p	P2RX7	Functional MTI	MIRT004357	Flow, In situ hybridization, Luciferase reporter assay, qRT-PCR, Western blot	24312495
hsa-miR-150-5p	MUC4	Functional MTI	MIRT006115	Luciferase reporter assay, qRT-PCR, Western blot	21983127
hsa-miR-150-5p	CXCR4	Functional MTI	MIRT006668	FACS, Western blot	22039399
hsa-miR-150-5p	NOTCH3	Functional MTI	MIRT007016	Luciferase reporter assay	21551231
hsa-miR-150-5p	ZEB1	Functional MTI	MIRT006984	Luciferase reporter assay, qRT-PCR, Western blot	23013135
hsa-miR-150-5p	ZEB1	Functional MTI	MIRT006984	ChIP-seq, Flow, Immunofluorescence, Luciferase reporter assay, qRT-PCR, Western blot	23765923
hsa-miR-150-5p	FLT3	Functional MTI	MIRT007053	Luciferase reporter assay	23079661
hsa-miR-150-5p	EP300	Functional MTI	MIRT007087	Luciferase reporter assay	23211718
hsa-miR-150-5p	EP300	Functional MTI	MIRT007087	Luciferase reporter assay, qRT-PCR, Western blot	27285761
hsa-miR-150-5p	TP53	Functional MTI	MIRT052652	Luciferase reporter assay, Western blot	23747308
hsa-miR-150-5p	TP53	Functional MTI	MIRT052652	Luciferase reporter assay, qRT-PCR	23670238
hsa-miR-150-5p	BIRC5	Functional MTI	MIRT054633	Immunohistochemistry, In situ hybridization, Microarray, qRT-PCR, Western blot	24613688
hsa-miR-150-5p	SRCIN1	Functional MTI	MIRT054852	Luciferase reporter assay, qRT-PCR, Western blot	24456795
hsa-miR-150-5p	CBL	Functional MTI	MIRT054868	Luciferase reporter assay, Microarray, qRT-PCR	23604034
hsa-miR-150-5p	COL4A4	Functional MTI	MIRT437823	Luciferase reporter assay	23604143
hsa-miR-150-5p	SP1	Functional MTI	MIRT437824	Luciferase reporter assay	23604143
hsa-miR-150-5p	SP1	Functional MTI	MIRT437824	Luciferase reporter assay, qRT-PCR, Western blot	28099946
hsa-miR-150-5p	CISH	Functional MTI	MIRT437917	Luciferase reporter assay, Western blot	23723424

hsa-miR-150-5p	CCR6	Functional MTI	MIRT438120	ELISA, Immunohistochemistry, Luciferase reporter assay, Northern blot, qRT-PCR, Western blot	24385540
hsa-miR-484	ZEB1	Functional MTI	MIRT734111	GFP reporter assay, Western blot	28286418
hsa-miR-484	FIS1	Functional MTI	MIRT006714	ChIP-seq, Immunoblot, LacZ reporter assay, qRT-PCR	22510686
hsa-miR-484	SMAD2	Functional MTI	MIRT042141	GFP reporter assay, Western blot	28286418
hsa-miR-93-5p	RHOC	Functional MTI	MIRT554752	Luciferase reporter assay	25649143
hsa-miR-93-5p	ABCA1	Functional MTI	MIRT728415	ELISA, Luciferase reporter assay, qRT-PCR, Western blot	25785043
hsa-miR-93-5p	RPS6KA4	Functional MTI	MIRT732756	Luciferase reporter assay, Next Generation Sequencing (NGS), qRT-PCR, Western blot	27350436
hsa-miR-93-5p	PDCD4	Functional MTI	MIRT733146	Luciferase reporter assay, qRT-PCR, Western blot	27021515
hsa-miR-93-5p	ANG	Functional MTI	MIRT733293	Luciferase reporter assay, Western blot	28401709
hsa-miR-93-5p	PTENP1	Functional MTI	MIRT734080	Luciferase reporter assay, qRT-PCR	28212532
hsa-miR-93-5p	ZNRF3	Functional MTI	MIRT734875	Luciferase reporter assay, qRT-PCR, Western blot	26423400
hsa-miR-93-5p	FOXO3	Functional MTI	MIRT735009	Luciferase reporter assay	25823655
hsa-miR-93-5p	MMP3	Functional MTI	MIRT735341	Luciferase reporter assay, qRT-PCR, Western blot	26175939
hsa-miR-93-5p	TP53INP1	Functional MTI	MIRT000021	Luciferase reporter assay, Western blot	18974142
hsa-miR-93-5p	FOXA1	Functional MTI	MIRT735529	Luciferase reporter assay, qRT-PCR, Western blot	27829043
hsa-miR-93-5p	CDKN1A	Functional MTI	MIRT000577	Luciferase reporter assay, Microarray, Western blot	18328430
hsa-miR-93-5p	CDKN1A	Functional MTI	MIRT000577	qRT-PCR, Luciferase reporter assay, Western blot	19153141
hsa-miR-93-5p	CDKN1A	Functional MTI	MIRT000577	qRT-PCR, Luciferase reporter assay, Western blot	20190813
hsa-miR-93-5p	CDKN1A	Functional MTI	MIRT000577	Luciferase reporter assay, Western blot	27185265
hsa-miR-93-5p	CDKN1A	Functional MTI	MIRT000577	qRT-PCR, Western blot	28356951
hsa-miR-93-5p	E2F1	Functional MTI	MIRT002472	Luciferase reporter assay, Western blot	19486339
hsa-miR-93-5p	E2F1	Functional MTI	MIRT002472	Luciferase reporter assay, Microarray, Western blot	18328430
hsa-miR-93-5p	VEGFA	Functional MTI	MIRT004055	ELISA, Luciferase reporter assay	18320040
hsa-miR-93-5p	ITGB8	Functional MTI	MIRT004104	Luciferase reporter assay, qRT-PCR, Western blot	20956944
hsa-miR-93-5p	VEGFA	Functional MTI	MIRT004055	Luciferase reporter assay, qRT-PCR, Western blot	26175939
hsa-miR-93-5p	TUSC2	Functional MTI	MIRT004410	Luciferase reporter assay, Western blot	19671678
hsa-miR-93-5p	KAT2B	Functional MTI	MIRT004332	Western blot, Luciferase reporter assay	18728182
hsa-miR-93-5p	PTEN	Functional MTI	MIRT006216	GFP reporter assay, qRT-PCR, Western blot	22465665
hsa-miR-93-5p	PTEN	Functional MTI	MIRT006216	Luciferase reporter assay	27119510
hsa-miR-93-5p	PTEN	Functional MTI	MIRT006216	Luciferase reporter assay, qRT-PCR, Western blot	26087719
hsa-miR-93-5p	PTEN	Functional MTI	MIRT006216	Luciferase reporter assay	25823655
hsa-miR-93-5p	PURA	Functional MTI	MIRT007003	Luciferase reporter assay	22835829
hsa-miR-93-5p	STK11	Functional MTI	MIRT028122	Luciferase reporter assay	28382150
hsa-miR-93-5p	LATS2	Functional MTI	MIRT007185	Luciferase reporter assay, qRT-PCR, Western blot	23111389
hsa-miR-93-5p	RAB11FIP1	Functional MTI	MIRT027958	Immunohistochemistry, Luciferase reporter assay, qRT-PCR, Western blot	27279231
hsa-miR-93-5p	CERS2	Functional MTI	MIRT028158	Western blot	27099514

hsa-miR-93-5p	PHLPP2	Functional MTI	MIRT028173	Luciferase reporter assay	25823655
hsa-miR-93-5p	TGFBR2	Functional MTI	MIRT027974	ELISA, Immunohistochemistry, Luciferase reporter assay, Microarray, qRT-PCR, Western blot	24606633
hsa-miR-93-5p	SLC2A4	Functional MTI	MIRT053085	Luciferase reporter assay, qRT-PCR, Western blot	23493574
hsa-miR-93-5p	ATG16L1	Functional MTI	MIRT053624	Immunofluorescence, Immunohistochemistry, In situ hybridization, Luciferase reporter assay, Western blot	24036151
hsa-miR-93-5p	DAB2	Functional MTI	MIRT053634	Flow, Luciferase reporter assay, Microarray, qRT-PCR, Western blot, colony formation	24037530
hsa-miR-93-5p	DAB2	Functional MTI	MIRT053634	Luciferase reporter assay, qRT-PCR, Western blot	25892549
hsa-miR-93-5p	SMAD7	Functional MTI	MIRT053874	Luciferase reporter assay, qRT-PCR, Western blot	22286770
hsa-miR-93-5p	ZBTB4	Functional MTI	MIRT054255	Luciferase reporter assay, qRT-PCR, Western blot	22752225
hsa-miR-93-5p	CXCL8	Functional MTI	MIRT438087	Luciferase reporter assay, qRT-PCR	24433094

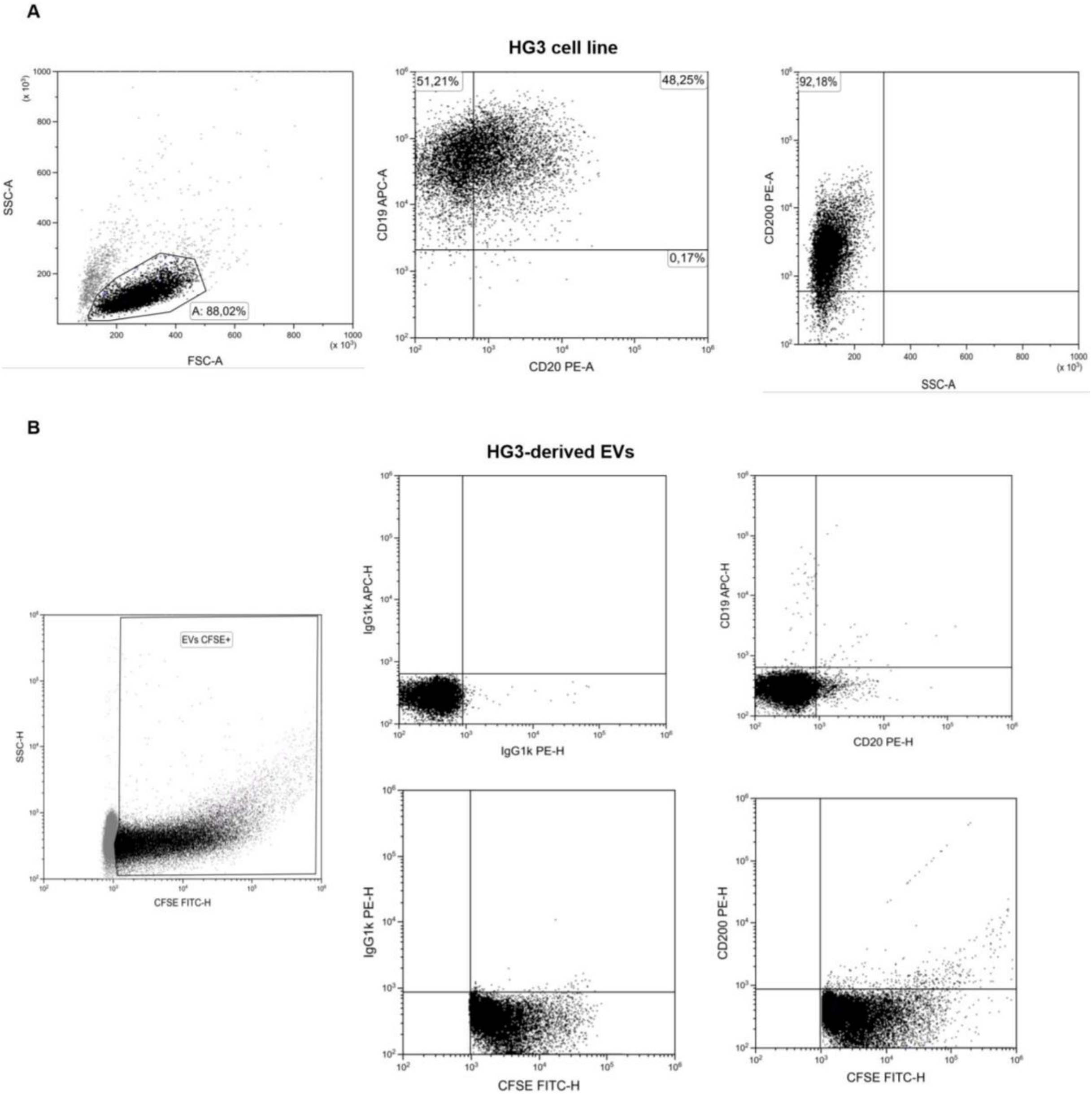

Figure S1. Dot plots of flow cytometric phenotypic analysis of (A) CLL cell line, HG3, and (B) HG3-derived EVs. Both HG3 cells and CFSE-labeled EVs were stained for CD19 APC, CD20 PE, CD200 PE, and their respective isotype (IgG1k PE or APC).

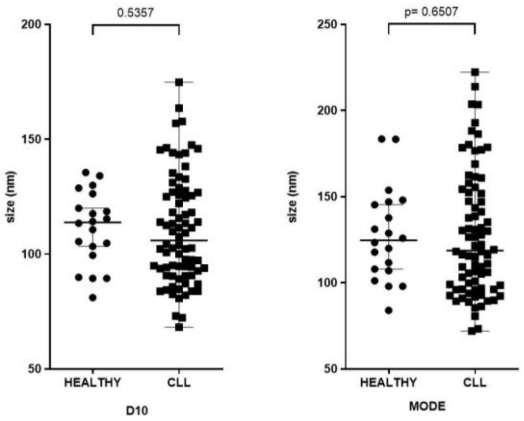

Figure S2. Dot plots of D10 and mode in EVs from healthy subjects compared to EVs from CLL patients. Each symbol represents a single subject, and horizontal bars represent median values with 95% CI.

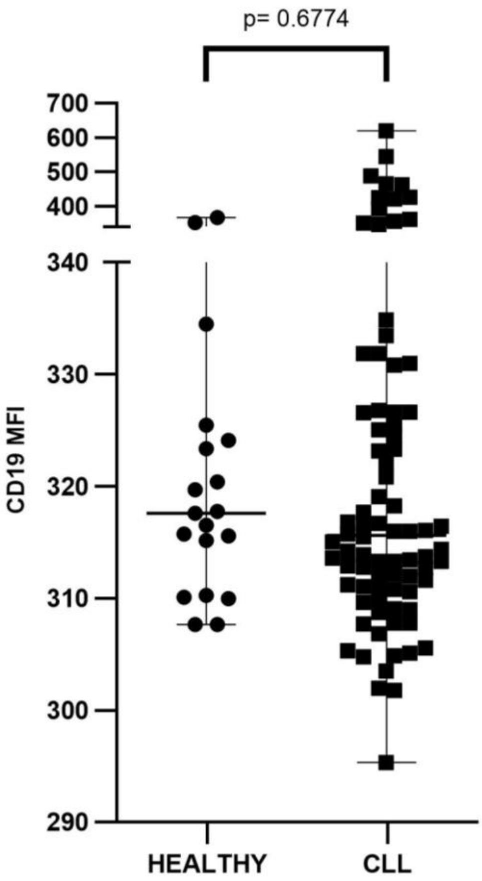

Figure S3. Dot plots of CD19 MFI in EVs from healthy subjects compared to EVs from CLL patients. Each symbol represents a single sample, and horizontal bars represent median values with 95% CI.

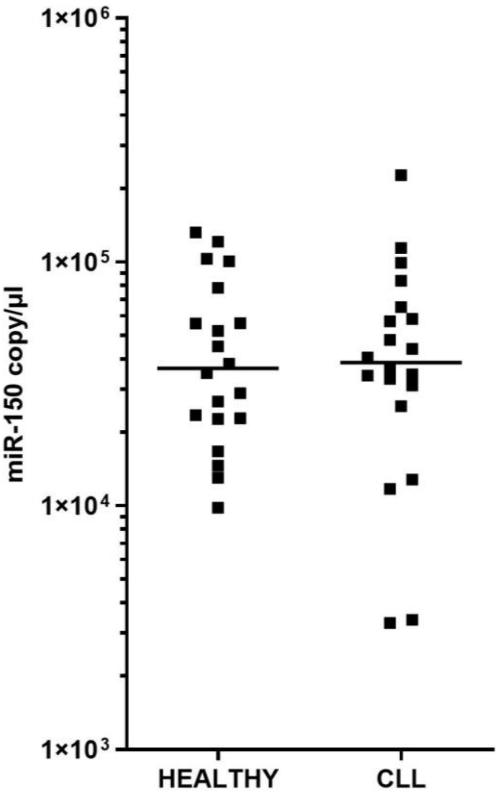

Figure S4. Dot plot of EV miR-150-5p level comparison between healthy subjects and CLL. Each symbol represents a single sample, and horizontal bars represent median values.

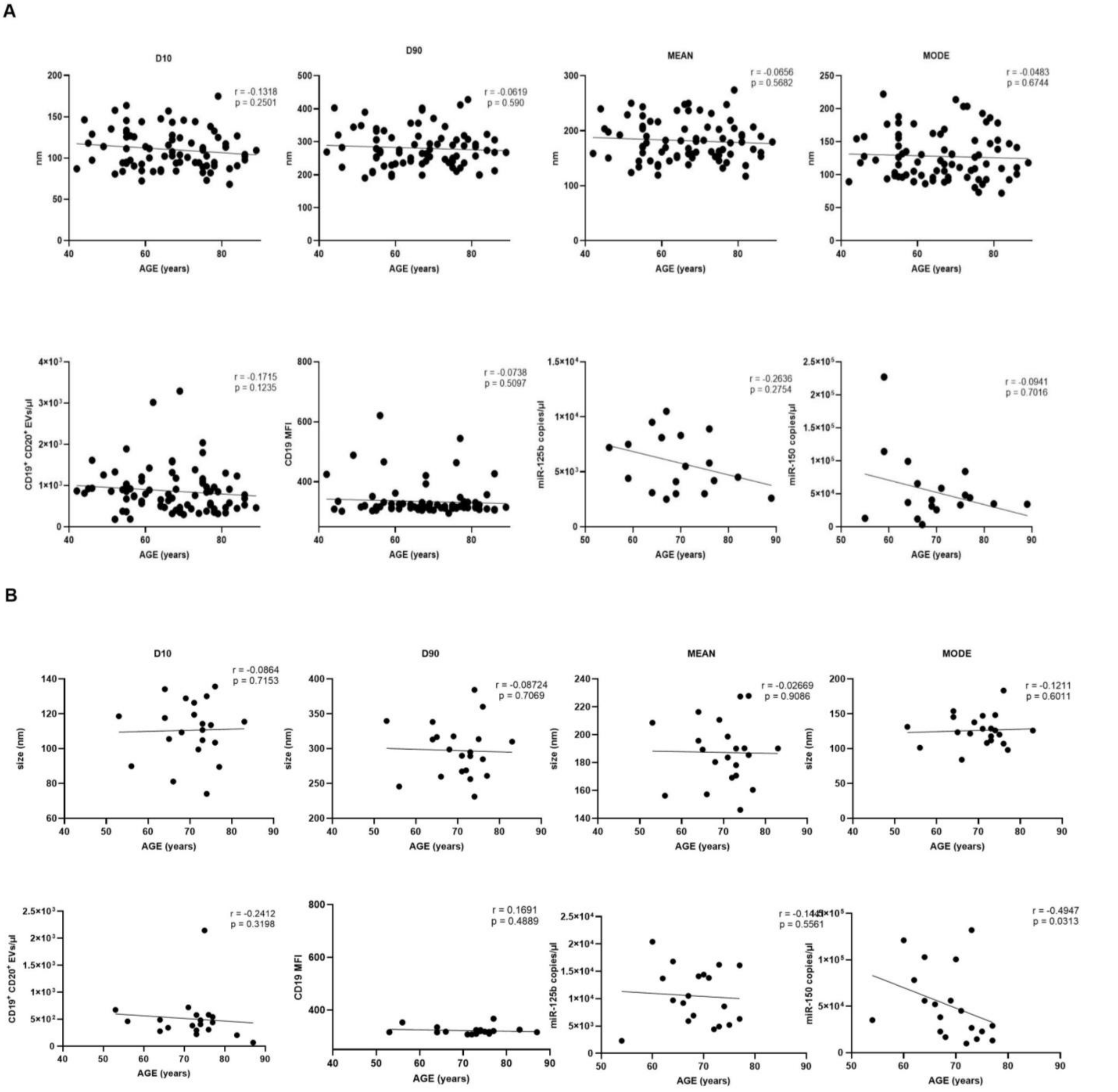

Figure S5. Dot plot correlations between (A) CLL patient age and size (D10, D90, mean, and mode), CD19⁺/CD20⁺ EVs/μL, CD19 MFI, EV miR-125b-5p, and EV miR-150-5p expression. R and p are indicated on plots. (B) Healthy subject age and concentration (EV/μL), size (D10, D90, mean, and mode), CD20⁺ EVs/μL, CD19⁺ EVs/μL, CD200⁺ EVs/μL, CD19⁺/CD20⁺ EVs/μL, CD20 MFI, CD19 MFI, CD200 MFI, EV miR-93, EV miR-125b-5p, EV miR-150-5p, and EV miR-484 expression.

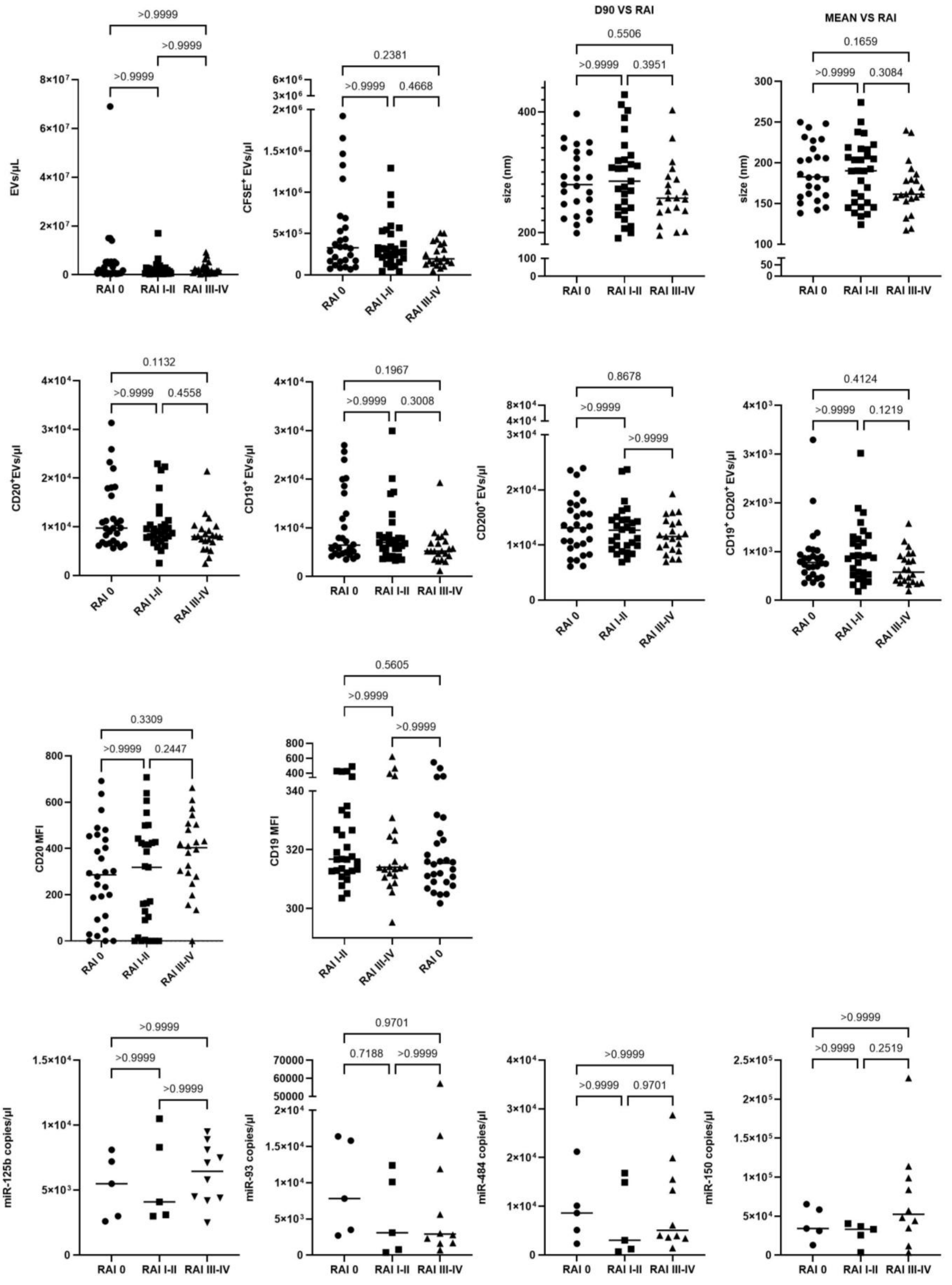

Figure S6. Plots of the trend of EV parameters (concentration by NTA and cytometer, D10, mean, CD20+ EVs/µL, CD19+ EVs/µL, CD200+ EVs/µL, CD19+/CD20+ EVs/µL, CD20 MFI, CD19 MFI, EV miR-125b-5p, EV miR-93, EV miR-484, and EV miR-150-5p amount (copies/µL) in CLL patients according to Rai stage.

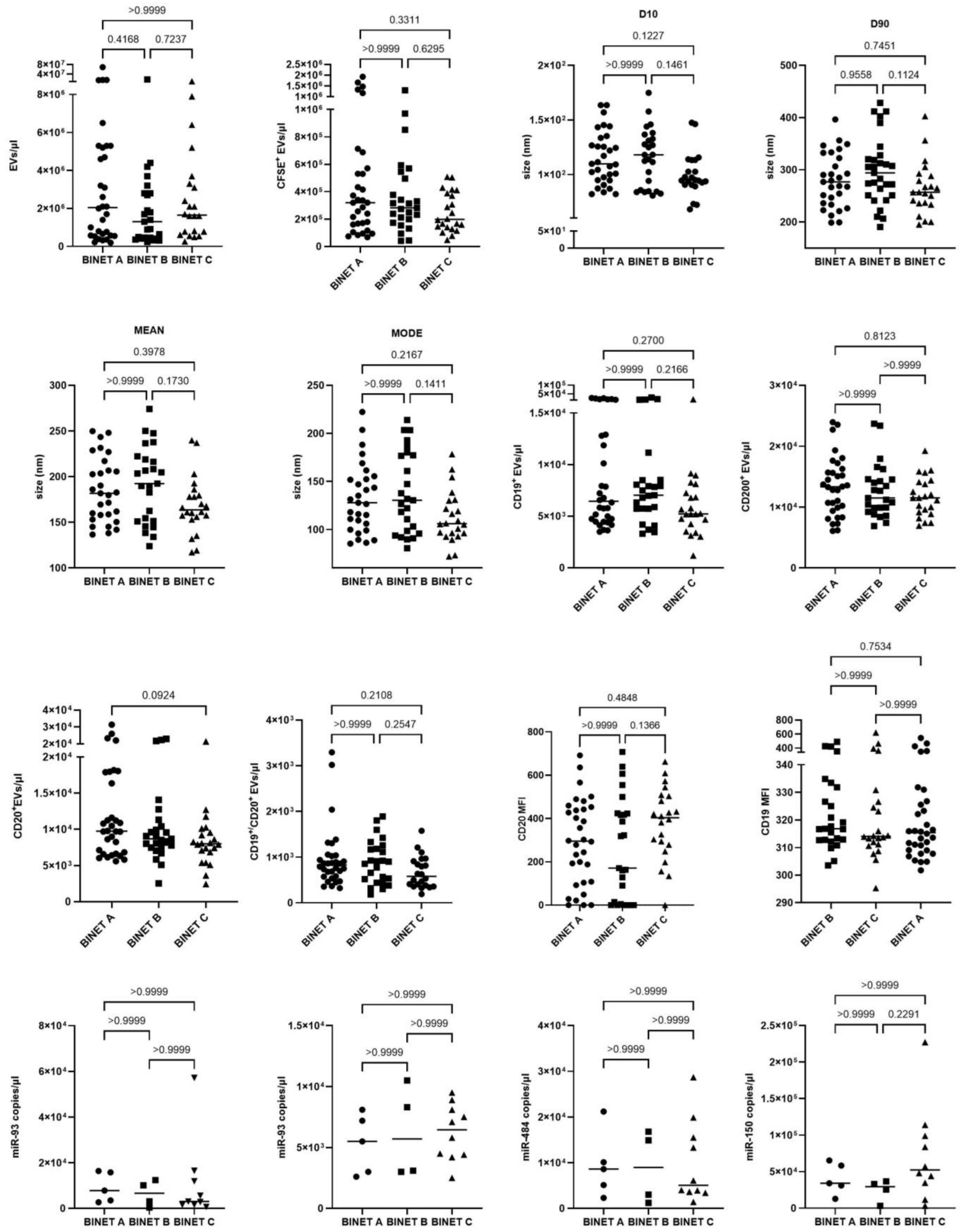

Figure S7. Plots of the trend of EV parameters (concentration by NTA and cytometer, D10, D90, mean, mode, CD19+ EVs/µL, CD200+ EVs/µL, CD19+/CD20+ EVs/µL, CD20 MFI, CD19 MFI, EV miR-93, EV miR-125b-5p, EV miR-484, and EV miR-150-5p amount (copies/µL) in CLL patients according to BINET stage.

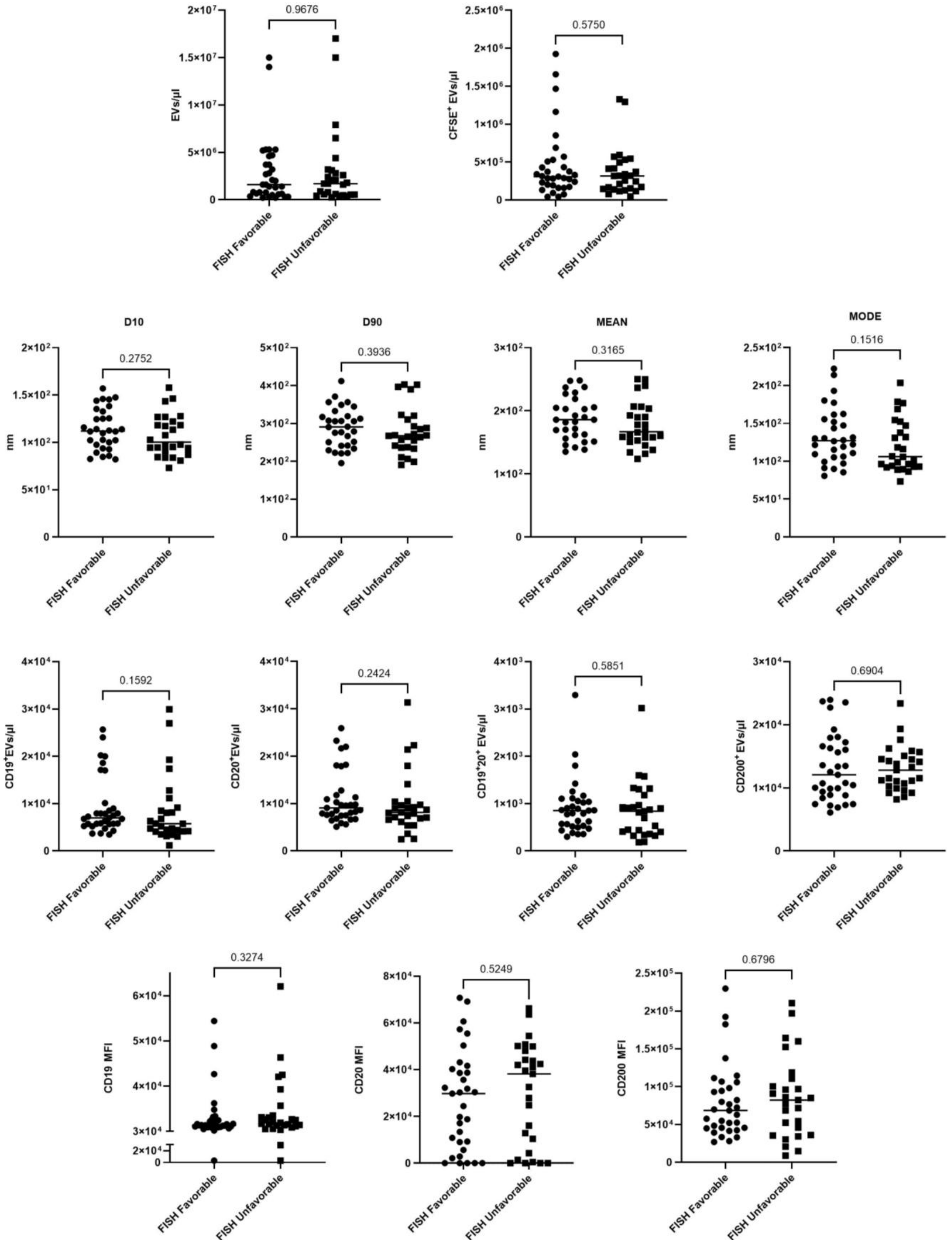

Figure S8. Dot plot of EV parameter (concentration by NTA and cytometer, D10, D90, mean, mode, $CD19^+$ EVs/μL, $CD20^+$ EVs/μL, $CD19^+$/$CD20^+$ EVs/μL, $CD200^+$ EVs/μL, CD19 MFI, CD20 MFI, and CD200 MFI EV) comparison between CLL patients with favorable FISH and unfavorable ones. Each symbol represents a single sample, and horizontal bars represent median values. Statistical analyses are reported on plots.

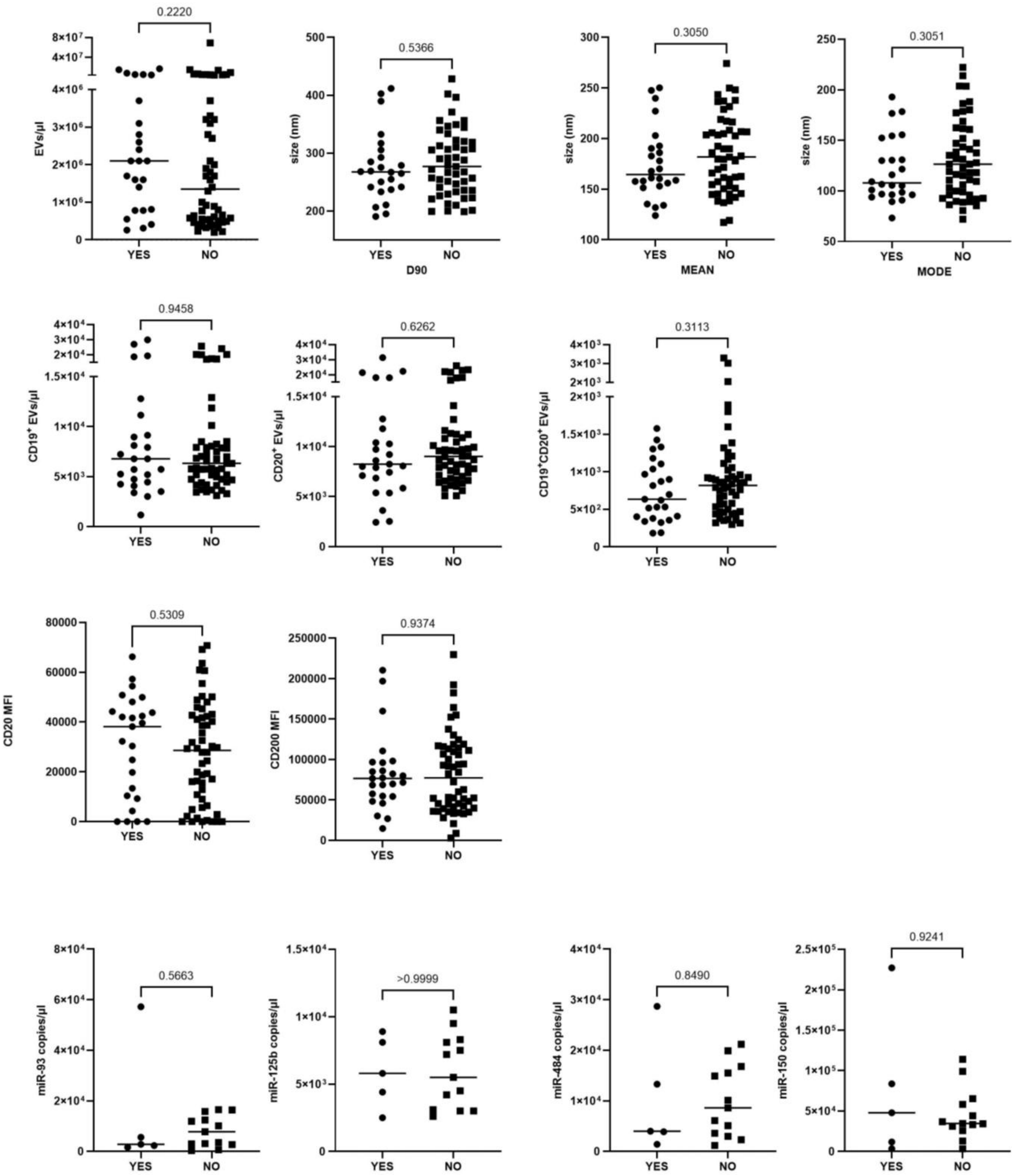

Figure S9. Dot plot of EV parameter comparison (concentration by NTA, D90, mean, mode, CD19+ EVs/μL, CD20+ EVs/μL, CD19+/CD20+ EVs/μL, CD20 MFI, and CD200 MFI EV, EV miR-93, EV miR-125b-5p, EV miR-484, and EV miR-150-5p amount (copies/μL)) between CLL patients who will be treated (YES) versus patients who will not be treated (NO). Each symbol represents a single sample, and horizontal bars represent median values. Statistical analyses are reported on plots.
